# Comparative efficacy of various art therapies for patients with dementia: A network meta-analysis of randomized controlled trials

**DOI:** 10.3389/fpsyt.2023.1072066

**Published:** 2023-01-25

**Authors:** Qian Liu, Fang Wang, Lixia Tan, Li Liu, Hong Cheng, Xiuying Hu

**Affiliations:** ^1^Innovation Center of Nursing Research, Nursing Key Laboratory of Sichuan, West China Hospital/West China School of Nursing, Sichuan University, Chengdu, China; ^2^School of Automation Engineering, University of Electronic Science and Technology of China, Chengdu, China

**Keywords:** art therapy, dementia, non-pharmaceutical therapy, network meta-analysis, older

## Abstract

**Background:**

Dementia have brought great challenges to patients, families and society. Numerous art therapies for patients with dementia have been developed in recent years. However, it is still unclear which art therapy represents the optimal strategy for promoting physical and mental health.

**Objectives:**

To compare the efficacy of various art therapies in improving cognitive function, activity of daily living, depression, anxiety, agitation behavior and quality of life, and rank the art therapies for practice consideration.

**Methods:**

A comprehensive literature search was performed in eight electronic databases from their inception to April 2022. Two authors independently completed study selection, data extraction, and assessed methodological quality according to the revised version of the Cochrane tool (RoB 2). Comparative evaluation of different art therapies’ effect was performed by conducting network meta-analysis. The study protocol was registered at PROSPERO.

**Results:**

A total of 39 randomized controlled trials involving 2801 participants were included. Calligraphy therapy (MD = 4.39) and reminiscence therapy (MD = 2.53) significantly improved cognitive function compared with the usual care, and reminiscence therapy (MD = 1.75) significantly enhanced cognitive function compared with music therapy. Horticultural therapy significantly decreased agitation behavior compared with the usual care (MD = −31.34), music therapy (MD = −26.66), reading therapy (MD = −28.44) and reminiscence therapy (MD = −27.32). In addition, calligraphy therapy (MD = 9.00) improved quality of life compared with the usual care.

**Conclusion:**

Calligraphy therapy might be the most effective art therapy for improving cognitive function and quality of life. Horticultural therapy might be the best art therapy for decreasing agitation behavior. Health-care professionals could consider applying these art therapies to improve cognitive function, agitation behavior and quality of life in patients with dementia.

## Introduction

The world is facing the major public health problem of dementia (1, 2). As the population ages, the number of people with dementia is expected to increase in the coming decades (3). It is estimated that there are about 50 million people suffering from dementia in the world, and the number is predicted to increase to 152 million by 2050 (4). Dementia is a syndrome of acquired and progressive cognitive impairment, and the patients typically experience poor quality of life, related to a reduced threshold for handling stressful environmental influences as well as behavioral and psychological symptoms of dementia (5). However, cognitive impairment, pool quality of life, behavioral and psychological symptoms of dementia will bring huge disease burden to the patients’ families and the society. It is reported that dementia is the fifth largest contributor to the global burden of disease, and its annual global economic cost has surpassed 1 trillion USD and will be 9.12 trillion USD in 2050 (3, 6).

At present, there is still no recognized gold standard for the pharmacological intervention of psychological and behavioral disorders in patients with dementia (7), and drugs may produce different side effects. The growing burden of dementia and a continuing lack of effective treatment make it increasingly important to develop and examine the effectiveness of interventions (8, 9). Non-pharmacological interventions have become the first choice for treating and caring the patients with dementia due to simplicity, ease of operation and high safety (10). Among non-pharmacological interventions, various art therapies have been widely proved to be beneficial to patients with dementia, which are helpful to reduce the psychological and behavioral abnormalities and slow down the progress of cognitive impairment (11). Art therapy has distinct characteristics, using art as a method to diagnose, treat or rehabilitate diseases in the artistic interaction with patients (12). The forms of art therapy are diverse and include performing visual and creative arts and medical humanities, such as music, singing, dance, reading and poetry groups as well as museum/gallery art and collections, creative writing, life story narrative-reminiscence, painting, printmaking, collage, pottery, sewing, knitting, woodwork or gardening (13). However, there is no evidence to prove which art therapy has the best effect on improving cognitive function, quality of life, and psychological and behavioral symptoms of patients with dementia. Network meta-analysis is developed from traditional meta-analysis, which can synthesize direct and indirect evidence and compare multiple interventions to provide greater precision. Therefore, the main purpose of this study was to compare the effects of different art therapies, so as to offer evidence-based information to help clinical decision-making.

## Materials and methods

This network meta-analysis was conducted according to the Preferred Reporting Items for Systematic Review and Meta-Analysis (PRISMA) guidelines extension statement for network meta-analysis (14). The protocol of this network meta-analysis was registered at PROSPERO (number: CRD4202 2314653).

### Data sources and searches

We searched eight databases, including Cochrane Central Register of Controlled Trials, PubMed, Web of Science, ScienceDirect, China BioMedical Literature Database, China National Knowledge Infrastructure, VIP Database, and Wanfang Database from their inception until April 2022 by using combination of the following keywords: dement*, Alzheimer*, arteriosclerotic encephalopathy, binswanger, vascular dementia, dementia with Lewy bodies, music*, singing, dance*, painting, drawing, collage, clay, theatre, drama, reading, poetry, woodwork, garden*, horticultural, handwriting, penmanship, calligraphy, ceramics, pottery, writing, sculpture, carving, narrative, reminiscence, printmaking, sewing, knitting, museum, gallery, and randomized controlled trial. Additionally, we reviewed the reference lists of eligible articles and reviews for additional eligible studies.

### Eligibility criteria

We included randomized controlled trials meeting all of the following criteria: (1) Participants: diagnosed with dementia by using the 4th edition Text Revision of Diagnostic and Statistical Manual of Mental Disorders (DSM-IV-TR) or probable Alzheimer’s disease (15); (2) Intervention: any kinds of art therapies, including but not restricted to music therapy, dancing therapy, reading therapy, painting therapy, horticultural therapy, reminiscence therapy, calligraphy therapy, and usual care for the experimental group or the control group; (3) Outcome: cognitive function, activity of daily living, depression, anxiety, agitation behavior or quality of life; (4) Language: written in English or Chinese; amd (5) Follow-up time: longer than four weeks. We excluded the studies: (1) multi-component intervention except any art therapy combined with a usual care; (2) participants with other major disease, such as malignant tumor and organ failure; and (3) cluster randomized controlled trials and cross-over randomized controlled trials.

### Study selection and data extraction

All retrieved studies will be imported into NoteExpress software to download references and remove the duplicates. Two reviewers independently screened the titles and abstracts for eligible studies and the preliminary results was cross-checked. Discrepancies were resolved through group discussions with a third reviewer.

For each study, two reviewers independently extracted the data, including the author (s), year of publication, country, sample size, characteristics of patients (gender and age), disease, intervention, outcome and measurement tool. If a study reported more than one post-treatment outcome scores, only the latest assessment following the conclusion of the intervention was used. Discrepancies were resolved by discussing with a third reviewer.

### Risk-of-bias assessment

The same two reviewers assessed the quality of each study by using the revised version of the Cochrane tool (RoB 2) (16), with any disagreement being resolved by consensus. This tool examined five domains (bias arising from the randomization process, bias due to deviations from intended interventions, bias due to missing outcome data, bias in the measurement of the outcome and bias in the selection of the reported result). The overall risk of bias judgment would be “low risk”, “some concerns” or “high risk.”

### Statistical analysis

We used mean difference (MD) and its 95% credible interval (CrI) to describe the results of continuous data. Considering the expected heterogeneity between studies, we conducted random-effects network meta-analyses for each outcome within a Bayesian framework by using Markov Chains Monte Carlo in R software (version 4.1.3). The relative efficacy of art therapies was estimated in accordance with the surface under the cumulative ranking curve (SUCRA) values. The network plots for each outcome were drawn in Stata software (version 15.0). The Egger’s test was used to evaluate the presence of publication bias in Stata software (version 15.0) when more than nine studies were included in analysis (17). The node-splitting analysis was used to assess the inconsistency if the outcome was a closed loop. Heterogeneity was evaluated with the *I*^2^statistics, and the *I*^2^ values were considered as none (0%), low (25%), moderate (50%) and high (75%) (18). To conduct the sensitivity analyses, we removed the study with the smallest sample size for each outcome. And then, the same methods to conduct Bayesian network analysis were repeated. The quality of evidence was evaluated by two authors based on the Grading of Recommendations Assessment, Development and Evaluation (GRADE) framework (19).

## Results

### Identification of relevant studies

**FIGURE 1:**
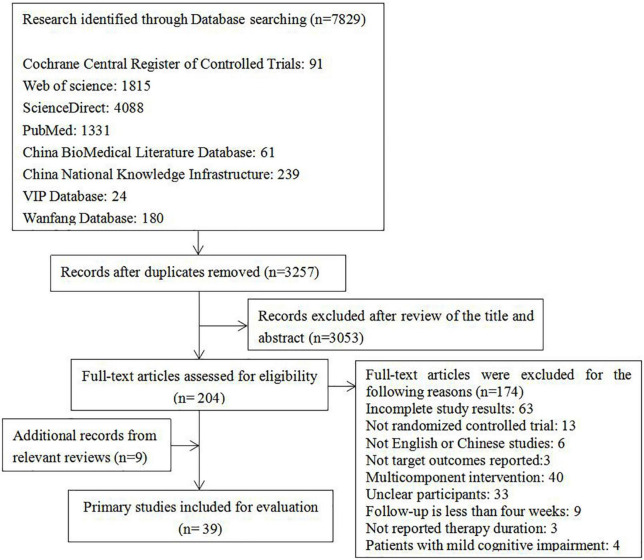
Study flow diagram.

As illustrated in Figure 1, a total of 7829 articles were identified and 39 articles were finally included in this review (Supplementary material 1). In total, these studies comprised 2,801 participants who received 6 types of art therapies combined with usual care or only the usual care of dementia. The specific interventions included music therapy, reading therapy, painting therapy, horticultural therapy, reminiscence therapy, calligraphy therapy and usual care. The definitions of these interventions are shown in Supplementary material 2. These studies included 6 outcomes, such as cognitive function, activity of daily living, depression, anxiety, agitation behavior and quality of life. The characteristics of the included studies are shown in Supplementary material 3.

### Risk-of-bias assessment

The quality of the 39 studies included in this network meta-analysis was evaluated in accordance with the RoB 2. Regarding five domains, 9 articles was rated as “low risk of bias,” 28 was “some concerns”, and 2 was “high risk of bias” (Supplementary material 4).

### Effects of art therapies on cognitive function

**FIGURE 2:**
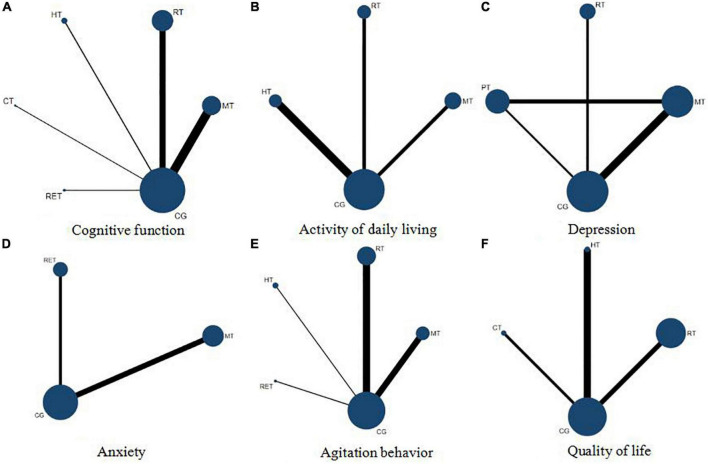
Network plots of cognitive function **(A)**, activity of daily living **(B)**, depression **(C)**, anxiety **(D)**, agitation behavior **(E)**, and quality of life **(F)**. MT, music therapy; RT, reminiscence therapy; HT, horticultural therapy; CT, calligraphy therapy; RET, reading therapy; PT, painting therapy; CG, control group with usual care.

Overall, 27 randomized controlled trials comprising 1,819 participants formed a non-closed loop (Figure 2A). The *I*^2^ value of both consistency model and inconsistency model was 3%. The effects of 6 interventions on cognitive function in patients with dementia are presented in Supplementary material 5a and Table 1A. Compared with the control groups (usual care), calligraphy therapy (MD = 4.39, 95% CrI 0.57-9.41) and reminiscence therapy (MD = 2.53, 95% CrI 1.37-3.68) significantly improved cognitive function. Furthermore, reminiscence therapy (MD = 1.75, 95% CrI 0.13-3.57) significantly enhanced cognitive function compared with music therapy. On the basis of the SUCRA values, the ranking of these art therapies is presented in Supplementary material 6 and calligraphy therapy was found to be superior to other art therapies.

**TABLE 1:** League tables for comparing different interventions.

(A). Cognitive function					
**CG**					
**–4.39 (–9.41, –0.57)**	**CT**				
–2.17 (–5.46, 1.04)	2.23 (–3.70, 8.16)	**HT**			
–0.78 (–2.15, 0.64)	3.61 (–1.51, 8.85)	1.38 (−2.10, 4.99)	**MT**		
0.35 (–4.40, 5.08)	4.74 (–2.13, 11.64)	2.52 (−3.20, 8.29)	1.14 (−3.84, 6.02)	**RET**	
−**2.53 (**−**3.68,**−**1.37)**	1.86 (−3.23, 7.01)	−0.36 (−3.76, 3.15)	−**1.75 (**−**3.57,**−**0.13)**	−2.88 (−7.73, 2.01)	**RT**
**(B). Activity of daily living**			
**CG**			
−1.86 (−14.86, 10.58)	**HT**		
2.26 (−8.33, 13.87)	4.13 (−12.00, 21.78)	**MT**	
−5.32 (−13.60, 3.76)	−3.44 (−18.27, 12.57)	−7.56 (−21.75, 6.50)	**RT**
**(C). Depression**			
**CG**			
0.14 (−3.40, 4.05)	**MT**		
0.95 (−3.02, 5.84)	0.83 (−3.52, 5.62)	**PT**	
5.03 (−0.63, 10.70)	4.90 (−2.04, 11.45)	4.08 (−3.52, 10.83)	**RT**
**(D). Anxiety**		
**CG**		
0.99 (−1.05, 2.98)	**MT**	
0.299 (−2.65, 3.25)	−0.69 (−4.23, 2.88)	**RET**
**(E). Agitation behavior**				
**CG**				
**31.34 (20.90, 41.60)**	**HT**			
4.68 (−0.75, 9.18)	−**26.66 (**−**38.60,**−**15.62)**	**MT**		
2.88 (−7.66, 13.30)	−**28.44 (**−**43.20,**−**13.66)**	−1.80 (−12.95, 10.24)	**RET**	
4.00 (−0.66, 8.35)	−**27.32 (**−**38.73,**−**16.09)**	−0.67 (−7.02, 6.35)	1.14 (−10.46, 12.46)	**RT**
**(F). Quality of life**			
**CG**			
−**9.00 (**−**17.58,**−**0.38)**	**CT**		
−1.03 (−10.64, 8.61)	7.95 (−4.90, 20.92)	**HT**	
−2.55 (−8.55, 3.50)	6.45 (−4.02, 16.88)	−1.51 (−12.82, 9.75)	**RT**

MT, music therapy; RT, reminiscence therapy; HT, horticultural therapy; CT, calligraphy therapy; RET, reading therapy; PT, painting therapy; CG, control group with usual care. Bold values indicate statistical significance.

### Effects of art therapies on activity of daily living

In total, 6 randomized controlled trials comprising 664 participants formed a non-closed loop (Figure 2B). Both of the *I*^2^ value of consistency model and inconsistency model were 4%. The effects of 4 interventions on activity of daily living in patients with dementia are presented in Supplementary material 5b and Table 1B. There was no significant difference between these interventions.

### Effects of art therapies on depression

Overall, 5 randomized controlled trials involving 240 participants formed a closed loop (Figure 2C). The *I*^2^ value of consistency model and inconsistency model were 12 and 9%, respectively. The effects of 4 interventions on depression are presented in Supplementary material 5c and Table 1C. There was no significant difference between the 4 interventions.

### Effects of art therapies on anxiety

Only 3 randomized controlled trials comprising 179 participants were analyzed for their efficacy toward anxiety and formed a non-closed loop (Figure 2D). The *I*^2^ value of consistency model and inconsistency model were 9 and 8%, respectively. The effects of 3 interventions on anxiety are presented in Supplementary material 5d and Table 1D. Between these studies, there was no significant difference.

### Effects of art therapies on agitation behavior

Overall, 12 randomized controlled trials involving 1240 participants were assessed for their efficacy in reducing agitation behavior. A non-closed loop was formed (Figure 2E). The I^2^ value of both consistency model and inconsistency model was 12%. The meta-analysis results included 5 interventions are shown in Supplementary material 5e and Table 1E. Compared with the control groups (usual care), horticultural therapy (MD = −31.34, 95% CrI −41.60- −20.90) significantly decreased agitation behavior. In addition, horticultural therapy significantly decreased agitation behavior compared with music therapy (MD = −26.66, 95% CrI −38.60- −15.62), reading therapy (MD = −28.44, 95% CrI −43.20- −13.66), and reminiscence therapy (MD = –27.32, 95% CrI –38.73- –16.09). The SUCRA values are shown in Supplementary material 6, revealing that horticultural therapy was most likely to be ranked as the best art therapy for decreasing agitation behavior.

### Effects of art therapies on quality of life

On the whole, 4 randomized controlled trials involving 270 participants were assessed for the effects of 4 interventions on quality of life and formed a non-closed loop (Figure 2F). The I^2^ value of both consistency model and inconsistency model was 9%. The findings of meta-analysis are listed in Supplementary material 5f and Table 1F. Calligraphy therapy (MD = 9.00, 95% CrI 0.38-17.58) significantly improved quality of life compared with the control groups (usual care). The SUCRA values are shown in Supplementary material 6, indicating that calligraphy therapy was most likely to be ranked as the optimal art therapy.

### Publication bias

The publication bias of this study with the outcome index of cognitive function and agitation behavior was assessed. The corresponding continuous Pr > | t| values of cognitive function and agitation behavior were respectively 0.255 and 0.357, indicating that the publication bias was not obvious.

### Inconsistency, heterogeneity and sensitivity analysis

The node-splitting model was conducted for depression only. The results showed that the P values of music therapy vs. usual care, painting therapy vs. usual care, and painting therapy vs. music therapy were respectively 0.330, 0.331, and 0.331, indicating the inconsistency was not significant (all *P* > 0.05). The heterogeneity analysis showed high level (*I*^2^ = 82.35%) of cognitive function, high level (*I*^2^ = 94.57%) of activity of daily living, low level (*I*^2^ = 15.07%) of depression, low level (*I*^2^ = 0%) of anxiety, moderate level (*I*^2^ = 57.61%) of agitation behavior, and low level (*I*^2^ = 0%) of quality of life. Sensitivity analyses were performed by excluding the studies with the smallest sample size for each outcome. For quality of life, only one study applied calligraphy therapy which were with the smallest sample size, so we removed the study with the second smallest sample size. All the remaining studies were listed in Supplementary material 7, and the results are shown in Supplementary material 8. Few differences further reflected that the small simple size may not influence intervention effect estimates.

### Quality of evidence

In accordance with the GRADE framework, the limitations of study, imprecision, heterogeneity, inconsistency, indirectness, and publication bias were evaluated. The overall quality of the evidence was moderate for cognitive function, activity of daily living and agitation behavior, and was high for depression, anxiety and quality of life.

## Discussion

To our knowledge, this is the first network meta-analysis to compare the effects of different art therapies on cognitive function, activity of daily living, depression, anxiety, agitation behavior and quality of life in patients with dementia. Additionally, this study performed sensitivity analyses to ensure that the results are relatively robust. In these network meta-analyses including 39 randomized controlled trials with 2,806 participants, it is revealed that art therapies are beneficial to improve cognitive function and quality of life, and to reduce agitation behavior in patients with dementia.

The results of direct comparison demonstrated that calligraphy therapy is better than the usual care in improving cognitive function and quality of life in patients with dementia, and reminiscence therapy could significantly enhance cognitive function compared with the usual care. Similarly, other researches (20, 21) found that calligraphy therapy is effective for enhancing cognitive function and quality of life in patients with dementia, and a meta-analysis showed that Chinese calligraphy therapy could help improve cognitive function in patients with neuropsychiatric symptoms (22). This may be because that calligraphy therapy, as a branch of art therapy that involves visual–spatial patterning of characters, could help patients to concentrate and control their body to complete calligraphy movements, so as to promote cognitive function, physical relaxation, emotional stability and quality of life. Pérez-Sáez et al. (23) supported that reminiscence therapy may have beneficial effects on cognition of people with dementia compared with usual care, which may be related to that reminiscence therapy could arouse people to review past events, emotions and thoughts through a variety of tangible cues including photos, music, scenes and videos. It not only exercises the abilities to memory and recall, but also improves the speech function and social interaction ability in the process of telling, thus improving the cognitive function of patients with dementia. Moreover, the network meta-analysis based on Bayesian random-effects model showed that calligraphy therapy is the best art therapy for cognitive function. Reminiscence therapy and music therapy comes second and third, respectively. This outcome may be because compared with reminiscence therapy and music therapy, calligraphy therapy could combine physical, mental and personal processes, and integrate visual performance, spatial abilities and cognitive planning, so as to help patients remain cognitively fit for a longer period of time (24).

In terms of agitation behavior, 12 randomized controlled trials comprising 1240 participants were involved to evaluate the effects of different art therapies on reducing agitation behavior. Direct comparison showed that horticultural therapy is better than usual care to decrease agitation behavior in patients with dementia, which is similar to the results of another research (25). Additionally, recent systematic reviews demonstrated that including therapeutic gardens in care environments is beneficial for people with dementia in some aspects, such as agitation, behavior, stress levels and aggressiveness (26, 27). According to the results of the network meta-analysis and rank probability analysis, horticultural therapy was the most effective art therapy against agitation behavior, probably because that contacting with nature is important to improve psychophysical wellbeing and quality of life, promote positive affective states, and reduce stress (26, 27). Furthermore, contacting with plants could stimulate the senses of patients with dementia to enhance the ability to perceive external things, and formulating appropriate gardening tasks also could promote the ability to think. Some gardening activities require group cooperation, which can enhance the patients’ expression ability and social skills and help them better adapt to society, so as to reduce agitation behavior.

However, there were some limitations that should be concerned. Firstly, although heterogeneity is a limitation in any network meta-analysis, differences in participants, study characteristics and sample size may restrict the internal validity of the findings. Secondly, the quality of included studies influence the quality of the findings. In this study, most of the randomized controlled trials (28 out of 39) did not clearly report the randomization process. Finally, the studies on some types of art therapy are relatively lacking. For example, in the network meta-analysis of cognitive function, there is only one study about reading therapy. Importantly, calligraphy therapy and horticulture therapy were considered the best art therapies in this network meta-analysis. However, the sample size of these studies of calligraphy therapy and horticulture therapy are small. Therefore, the future researches should pay more attention to conducting randomized controlled trials to confirm the effectiveness of these art therapies for better evidence.

## Conclusion

Six art therapies and usual care included in this present network meta-analysis had different advantages in improving cognitive function, agitation behavior and quality of life. Based on the results, calligraphy therapy might be the optimal art therapy to promote cognitive function and quality of life, and horticultural therapy is superior to other art therapies for reducing agitation behavior. Healthcare professionals should be encouraged to apply these art therapies to patients with dementia, and calligraphy therapy and horticultural therapy should be incorporated as part of routine programs.

## Data availability statement

The original contributions presented in this study are included in this article/Supplementary material, further inquiries can be directed to the corresponding author.

## Author contributions

QL: conceptualization, methodology, software, formal analysis, data curation, and writing – original draft. FW: conceptualization, supervision, and writing – review and editing. LT and LL: conceptualization and methodology. HC: writing – review and editing. XH: conceptualization, writing – review and editing, supervision, and project administration. All authors contributed to the article and approved the submitted version.
