# Supplementary material for: Comparative efficacy of various art therapies for patients with dementia: A network meta-analysis of randomized controlled trials
Source: Front Psychiatry. 2023 Jan 25;14:1072066. doi: 10.3389/fpsyt.2023.1072066 (PMC9905634; doi:10.3389/fpsyt.2023.1072066)
Supplement: Supplementary file 1 [file Data_Sheet_1.docx]

Supplementary Material

Contents

Supplementary material 1: Lists of 39 included studies

Supplementary material 2: The definitions of included art therapies

Supplementary material 3: Characteristics of 39 studies included in this study

Supplementary material 4: Risk of bias for included studies

Supplementary material 5: Forest plots of network meta-analysis results for outcomes.

Supplementary material 6: The SUCRA of different interventions

Supplementary material 7: Lists of included studies for sensitivity analysis

Supplementary material 8: Results of sensitivity analysis

# Supplementary Material 1. Lists of 39 included studies

**Cognitive function**

1. Pedrinolla A, Tamburin S, Brasioli A, Sollima A, Fonte C, Muti E, et al. An Indoor Therapeutic Garden for Behavioral Symptoms in Alzheimer's Disease: A Randomized Controlled Trial. J Alzheimers Dis. 2019;71(3):813-823. doi: 10.3233/JAD-190394
2. Giovagnoli AR, Manfredi V, Schifano L, Paterlini C, Parente A, Tagliavini F. Combining drug and music therapy in patients with moderate Alzheimer's disease: a randomized study. Neurol Sci. 2018;39(6):1021-1028. doi: 10.1007/s10072-018-33 16-3
3. Gómez-Gallego M, Gómez-Gallego JC, Gallego-Mellado M, García-García J. Comparative Efficacy of Active Group Music Intervention versus Group Music Listening in Alzheimer's Disease. Int J Environ Res Public Health. 2021;18(15):8067. doi: 10.3390/ijerph18158067
4. Yang Y, Kwan RYC, Zhai HM, Xiong Y, Zhao T, Fang KL, Zhang HQ. Effect of horticultural therapy on apathy in nursing home residents with dementia: a pilot randomized controlled trial. Aging Ment Health. 2022;26(4):745-753. doi: 10.1080/13607863.2021.1907304
5. Nakamae T, Yotsumoto K, Tatsumi E, Hashimotoa T. Effects of Productive Activities with Reminiscence in Occupational Therapy for People with Dementia: A Pilot Randomized Controlled Study. Hong Kong Journal of Occupational Therapy. 2014;24(1):13-19.doi: 10.1016/j.hkjot.2014.01.003
6. Tadaka E, Kanagawa K. Effects of reminiscence group in elderly people with Alzheimer disease and vascular dementia in a community setting. Geriatr Gerontol Int. 2007; 7(2):167-173. doi: 10.1111/j.1447-0594.2007.00381.x
7. Van Bogaert P, Van Grinsven R, Tolson D, Wouters K, Engelborghs S, Van der Mussele S. Effects of SolCos model-based individual reminiscence on older adults with mild to moderate dementia due to Alzheimer disease: a pilot study. J Am Med Dir Assoc. 2013;14(7):528.e9-13. doi: 10.1016/j.jamda.2013.01.020
8. Wang JJ. Group reminiscence therapy for cognitive and affective function of demented elderly in Taiwan. Int J Geriatr Psychiatry. 2007;22(12):1235-40. doi: 10.1002/gps.1821
9. Pérez-Sáez E, Justo-Henriques SI, Alves Apóstolo JL. Multicenter randomized controlled trial of the effects of individual reminiscence therapy on cognition, depression and quality of life: Analysis of a sample of older adults with Alzheimer's disease and vascular dementia. Clin Neuropsychol. 2021:1-22. doi: 10.1080/13854046.2021.1871962
10. Wu LF, Koo M. Randomized controlled trial of a six-week spiritual reminiscence intervention on hope, life satisfaction, and spiritual well-being in elderly with mild and moderate dementia. Int J Geriatr Psychiatry. 2016;31(2):120-7. doi: 10.1002/gps.4300
11. Duru Aşiret G, Kapucu S. The Effect of Reminiscence Therapy on Cognition, Depression, and Activities of Daily Living for Patients With Alzheimer Disease. J Geriatr Psychiatry Neurol. 2016;29(1):31-7. doi: 10.1177/0891988715598233
12. Lök N, Bademli K, Selçuk-Tosun A. The effect of reminiscence therapy on cognitive functions, depression, and quality of life in Alzheimer patients: Randomized controlled trial. Int J Geriatr Psychiatry. 2019;34(1):47-53. doi: 10.1002/gps.4980
13. İnel Manav A, Simsek N. The Effect of Reminiscence Therapy With Internet-Based Videos on Cognitive Status and Apathy of Older People With Mild Dementia. J Geriatr Psychiatry Neurol. 2019;32(2):104-113. doi: 10.1177/0891988718819864
14. Lyu J, Zhang J, Mu H, Li W, Champ M, Xiong Q, et al. The Effects of Music Therapy on Cognition, Psychiatric Symptoms, and Activities of Daily Living in Patients with Alzheimer's Disease. J Alzheimers Dis. 2018;64(4):1347-1358. doi: 10.3233/JAD-180183
15. Chu H, Yang CY, Lin Y, Ou KL, Lee TY, O'Brien AP, et al. The impact of group music therapy on depression and cognition in elderly persons with dementia: a randomized controlled study. Biol Res Nurs. 2014;16(2):209-17. doi: 10.1177/1099800413485410
16. Wang LY, Shen CZ. Intervention practice of senile dementia based on life story book.Chinese Journal of Gerontology. 2013;33(21):5239-5241. (in Chinese)
17. Ge ZX, Deng XL. Observation on rehabilitation effect of memory therapy in senile dementia patients.Journal of Nursing(China). 2013;20(03):63-65. (in Chinese) doi: 10.16460/j.issn1008-9969.2013.03.025.
18. Yuan LX. Effect of arithmetic or drawing writing on communication and cognitive function in patients with mild to moderate dementia. Guangzhou Medical University: Master Thesis, 2018. (in Chinese)
19. Liu LC, Liu Y. Effect of music therapy on senile dementia patients. Chinese Journal of Gerontology. 2017;37(05):1215-1216. (in Chinese)
20. Ito T, Meguro K, Akanuma K, Ishii H, Mori E. A randomized controlled trial of the group reminiscence approach in patients with vascular dementia. Dement Geriatr Cogn Disord. 2007;24(1):48-54. doi: 10.1159/000103631
21. Giovagnoli AR, Manfredi V, Parente A, Schifano L, Oliveri S, Avanzini G. Cognitive training in Alzheimer's disease: a controlled randomized study. Neurol Sci. 2017;38(8):1485-1493. doi: 10.1007/s10072-017-3003-9
22. Ceccato E, Vigato G, Bonetto C, Bevilacqua A, Pizziolo P, Crociani S, Zanfretta E, Pollini L, Caneva PA, Baldin L, Frongillo C, Signorini A, Demoro S, Barchi E. STAM protocol in dementia: a multicenter, single-blind, randomized, and controlled trial. Am J Alzheimers Dis Other Demen. 2012;27(5):301-10. doi: 10.1177/1533317512452038
23. Choi H M J . Songwriting oriented activities improve the cognitive functions of the aged with dementia. The Arts in Psychotherapy.2011. doi: 10.1016/j.aip.2011.07.002
24. Janata, P. Effects of Widespread and Frequent Personalized Music Programming on Agitation and Depression in Assisted Living Facility Residents With Alzheimer-Type Dementia. Music & Medicine. 2012;4(1):8-15. doi: 10.1177/1943862111430509
25. Du YQ, Zhao X. Effect of music nursing intervention on agitation behavior and cognitive function in patients with moderate and mild senile dementia. Chinese Journal of Geriatric Care. 2018;16(04):128-130. (in Chinese)
26. Tang Q, Zhou Y, Yang S, Thomas WKS, Smith GD, Yang Z, et al. Effect of music intervention on apathy in nursing home residents with dementia. Geriatr Nurs. 2018;39(4):471-476. doi: 10.1016/j.gerinurse.2018.02.003
27. Lyv JH, Gao T, Li M, Xie LJ, Li WJ, Jin WY, et al. The effect of music therapy on memory, language and psychological symptoms of patients with mild Alzheimer’s disease .Chinese Journal of Neurology. 2014;47(12):831-835. (in Chinese)

**Activity of daily living**

1. Pedrinolla A, Tamburin S, Brasioli A, Sollima A, Fonte C, Muti E, et al. An Indoor Therapeutic Garden for Behavioral Symptoms in Alzheimer's Disease: A Randomized Controlled Trial. J Alzheimers Dis. 2019;71(3):813-823. doi: 10.3233/JAD-190394
2. Gómez-Gallego M, Gómez-Gallego JC, Gallego-Mellado M, García-García J. Comparative Efficacy of Active Group Music Intervention versus Group Music Listening in Alzheimer's Disease. Int J Environ Res Public Health. 2021;18(15):8067. doi: 10.3390/ijerph18158067
3. Yang Y, Kwan RYC, Zhai HM, Xiong Y, Zhao T, Fang KL, Zhang HQ. Effect of horticultural therapy on apathy in nursing home residents with dementia: a pilot randomized controlled trial. Aging Ment Health. 2022;26(4):745-753. doi: 10.1080/13607863.2021.1907304
4. Li M, Lyu JH, Zhang Y, Gao ML, Li R, Mao PX, et al. Efficacy of Group Reminiscence Therapy on Cognition, Depression, Neuropsychiatric Symptoms, and Activities of Daily Living for Patients With Alzheimer Disease. J Geriatr Psychiatry Neurol. 2020;33(5):272-281. doi: 10.1177/0891988719882099
5. Lyu J, Zhang J, Mu H, Li W, Champ M, Xiong Q, et al. The Effects of Music Therapy on Cognition, Psychiatric Symptoms, and Activities of Daily Living in Patients with Alzheimer's Disease. J Alzheimers Dis. 2018;64(4):1347-1358. doi: 10.3233/JAD-180183
6. Ge ZX, Deng XL. Observation on rehabilitation effect of memory therapy in senile dementia patients.Journal of Nursing(China). 2013;20(03):63-65. (in Chinese) doi: 10.16460/j.issn1008-9969.2013.03.025.

**Depression**

1. Pongan E, Tillmann B, Leveque Y, Trombert B, Getenet JC, et al. Can Musical or Painting Interventions Improve Chronic Pain, Mood, Quality of Life, and Cognition in Patients with Mild Alzheimer's Disease? Evidence from a Randomized Controlled Trial. J Alzheimers Dis. 2017;60(2):663-677. doi: 10.3233/JAD-170410
2. Hattori H, Hattori C, Hokao C, Mizushima K, Mase T. Controlled study on the cognitive and psychological effect of coloring and drawing in mild Alzheimer's disease patients. Geriatr Gerontol Int. 2011;11(4):431-7. doi: 10.1111/j.1447-0594.2011.00698.x
3. Guétin S, Portet F, Picot MC, Pommié C, Messaoudi M, Djabelkir L, et al. Effect of music therapy on anxiety and depression in patients with Alzheimer's type dementia: randomised, controlled study. Dement Geriatr Cogn Disord. 2009;28(1):36-46. doi: 10.1159/000229024
4. Duru Aşiret G, Kapucu S. The Effect of Reminiscence Therapy on Cognition, Depression, and Activities of Daily Living for Patients With Alzheimer Disease. J Geriatr Psychiatry Neurol. 2016;29(1):31-7. doi: 10.1177/0891988715598233
5. Ceccato E, Vigato G, Bonetto C, Bevilacqua A, Pizziolo P, Crociani S, Zanfretta E, Pollini L, Caneva PA, Baldin L, Frongillo C, Signorini A, Demoro S, Barchi E. STAM protocol in dementia: a multicenter, single-blind, randomized, and controlled trial. Am J Alzheimers Dis Other Demen. 2012;27(5):301-10. doi: 10.1177/1533317512452038

**Anxiety**

1. Sung HC, Lee WL, Li TL, Watson R. A group music intervention using percussion instruments with familiar music to reduce anxiety and agitation of institutionalized older adults with dementia. Int J Geriatr Psychiatry. 2012;27(6):621-7. doi: 10.1002/gps.2761
2. Sung HC, Chang AM, Lee WL. A preferred music listening intervention to reduce anxiety in older adults with dementia in nursing homes. J Clin Nurs. 2010;19(7-8):1056-64. doi: 10.1111/j.1365-2702.2009.03016.x
3. Goyal AR, Engedal K, Benth JŠ, Strøm BS. Effects of the Sonas Program on Anxiety and Depression in Nursing Home Residents with Dementia: A 6-Month Randomized Controlled Trial. Dement Geriatr Cogn Dis Extra. 2021;11(2):151-158. doi: 10.1159/000516804

**Agitation behavior**

1. Pedrinolla A, Tamburin S, Brasioli A, Sollima A, Fonte C, Muti E, et al. An Indoor Therapeutic Garden for Behavioral Symptoms in Alzheimer's Disease: A Randomized Controlled Trial. J Alzheimers Dis. 2019;71(3):813-823. doi: 10.3233/JAD-190394
2. Giovagnoli AR, Manfredi V, Schifano L, Paterlini C, Parente A, Tagliavini F. Combining drug and music therapy in patients with moderate Alzheimer's disease: a randomized study. Neurol Sci. 2018;39(6):1021-1028. doi: 10.1007/s10072-018-33 16-3
3. Gómez-Gallego M, Gómez-Gallego JC, Gallego-Mellado M, García-García J. Comparative Efficacy of Active Group Music Intervention versus Group Music Listening in Alzheimer's Disease. Int J Environ Res Public Health. 2021;18(15):8067. doi: 10.3390/ijerph18158067
4. Raglio A, Bellandi D, Baiardi P, Gianotti M, Ubezio MC, Zanacchi E, et al. Effect of Active Music Therapy and Individualized Listening to Music on Dementia: A Multicenter Randomized Controlled Trial. J Am Geriatr Soc. 2015;63(8):1534-9. doi: 10.1111/jgs.13558
5. Van Bogaert P, Van Grinsven R, Tolson D, Wouters K, Engelborghs S, Van der Mussele S. Effects of SolCos model-based individual reminiscence on older adults with mild to moderate dementia due to Alzheimer disease: a pilot study. J Am Med Dir Assoc. 2013;14(7):528.e9-13. doi: 10.1016/j.jamda.2013.01.020
6. Li M, Lyu JH, Zhang Y, Gao ML, Li R, Mao PX, et al. Efficacy of Group Reminiscence Therapy on Cognition, Depression, Neuropsychiatric Symptoms, and Activities of Daily Living for Patients With Alzheimer Disease. J Geriatr Psychiatry Neurol. 2020;33(5):272-281. doi: 10.1177/0891988719882099
7. Amieva H, Robert PH, Grandoulier AS, Meillon C, De Rotrou J, Andrieu S, et al. Group and individual cognitive therapies in Alzheimer's disease: the ETNA3 randomized trial. Int Psychogeriatr. 2016;28(5):707-17. doi: 10.1017/S1041610215001830
8. Lyu J, Zhang J, Mu H, Li W, Champ M, Xiong Q, et al. The Effects of Music Therapy on Cognition, Psychiatric Symptoms, and Activities of Daily Living in Patients with Alzheimer's Disease. J Alzheimers Dis. 2018;64(4):1347-1358. doi: 10.3233/JAD-180183
9. Elfrink TR, Ullrich C, Kunz M, Zuidema SU, Westerhof GJ. The Online Life Story Book: A randomized controlled trial on the effects of a digital reminiscence intervention for people with (very) mild dementia and their informal caregivers. PLoS One. 2021;16(9):e0256251. doi: 10.1371/journal.pone.0256251
10. Hatakeyama R, Fukushima K, Fukuoka Y, Satoh A, Kudoh H, Fujii M, Sasaki H. Personal home made digital video disk for patients with behavioral psychological symptoms of dementia. Geriatr Gerontol Int. 2010 Jul;10(3):272-4. doi: 10.1111/j.1447-0594.2010.00623.x
11. Li M, Lyu JH, Gao ML, Hao ZH, Ma L, Li WJ. The effect of reminiscence therapy on behavioral and psychological symptoms in people with mild and moderate dementia. Beijing Medical Journal. 2016;38(10):999-1002. (in Chinese) doi: 10.15932/j.0253-9713.2016.10.008
12. Lyv JH, Gao T, Li M, Xie LJ, Li WJ, Jin WY, et al. The effect of music therapy on memory, language and psychological symptoms of patients with mild Alzheimer’s disease .Chinese Journal of Neurology. 2014;47(12):831-835. (in Chinese)

**Quality of life**

1. Yang Y, Kwan RYC, Zhai HM, Xiong Y, Zhao T, Fang KL, Zhang HQ. Effect of horticultural therapy on apathy in nursing home residents with dementia: a pilot randomized controlled trial. Aging Ment Health. 2022;26(4):745-753. doi: 10.1080/13607863.2021.1907304
2. Pérez-Sáez E, Justo-Henriques SI, Alves Apóstolo JL. Multicenter randomized controlled trial of the effects of individual reminiscence therapy on cognition, depression and quality of life: Analysis of a sample of older adults with Alzheimer's disease and vascular dementia. Clin Neuropsychol. 2021:1-22. doi: 10.1080/13854046.2021.1871962
3. Lök N, Bademli K, Selçuk-Tosun A. The effect of reminiscence therapy on cognitive functions, depression, and quality of life in Alzheimer patients: Randomized controlled trial. Int J Geriatr Psychiatry. 2019;34(1):47-53. doi: 10.1002/gps.4980
4. Yuan LX. Effect of arithmetic or drawing writing on communication and cognitive function in patients with mild to moderate dementia. Guangzhou Medical University: Master Thesis, 2018. (in Chinese)

# Supplementary material 2. The definitions of included art therapies

1. Music therapy: Based on the practical function of music, music therapy is a method to treat diseases or promote physical and mental health by using music or music related experience according to the systematic treatment process (1).
2. Reading therapy: Reading therapy refers to the use of any literature that supports good mental health and meets people’s therapeutic or developmental needs (2-3).
3. Painting therapy: Painting therapy is one of the methods for psychological art treatment, which enables painters to present the repressed feelings and conflicts in the subconscious through the creative process of painting and using non-verbal tools, and obtain the expression and satisfaction in the process of painting, so as to achieve a good effect of diagnosis and treatment (4).
4. Horticultural therapy: Horticultural therapy is the engagement of a client in horticulture activities facilitated by a trained therapist to achieve specific and documented treatment goals (5).
5. Reminiscence therapy: Remembrance therapy refers to helping increase well-being, quality of life, and resilience to existing circumstances through the review and discussion of past activities, events and experiences, usually with the aid of tangible prompts (e.g. photographs, household and other familiar items from the past, music and archive sound recordings) (6). In this study, the tangible prompts should be music, photographs and familiar items from the past.
6. Calligraphy therapy: In this study, calligraphy therapy refers to Chinese calligraphy handwriting, which is a branch of art therapy and a culture-based exercise involving handwriting of Chinese characters with a brush, so as to activate and facilitate positive changes in the practitioner’s physiological, cognitive, and emotional well-being with proven therapeutic and rehabilitative success (7-8).
7. Usual care: All patients with dementia should received usual care in accordance with clinical guidance, including counselling for carers and families, a clinical assessment, prescription of symptomatic treatments, and some advice about drug, diet, activity, rehabilitation and complication prevention

References:

1. Gassner L, Geretsegger M, Mayer-Ferbas J. Effectiveness of music therapy for autism spectrum disorder, dementia, depression, insomnia and schizophrenia: update of systematic reviews. Eur J Public Health. 2022; 32(1):27-34. doi: 10.1093/eurpub/ckab042
2. Wang S, Cheung DSK, Bressington D, Li Y, Leung AYM. The Development of an Evidence-Based Telephone-Coached Bibliotherapy Protocol for Improving Dementia Caregiving Appraisal. Int J Environ Res Public Health. 2022;19(14):8731. doi: 10.3390/ijerph19148731
3. Malibiran R, Tariman JD, Amer K. Bibliotherapy: Appraisal of Evidence for Patients Diagnosed With Cancer. Clin J Oncol Nurs. 2018;22(4):377-380. doi: 10.1188/18.CJON.377-380
4. Yuan Z, Li B, Ye X, Zhu X. Systematic review and meta-analysis of the effects of group painting therapy on the negative emotions of depressed adolescent patients. Ann Palliat Med. 2021;10(10):10744-10755. doi: 10.21037/apm-21-2474
5. Siu AMH, Kam M, Mok I. Horticultural Therapy Program for People with Mental Illness: A Mixed-Method Evaluation. Int J Environ Res Public Health. 2020;17(3):711. doi: 10.3390/ijerph17030711
6. Woods B, O'Philbin L, Farrell EM, Spector AE, Orrell M. Reminiscence therapy for dementia. Cochrane Database Syst Rev. 2018;3(3):CD001120. doi: 10.1002/14651858.CD001120.pub3
7. Chu KY, Huang CY, Ouyang WC. Does Chinese calligraphy therapy reduce neuropsychiatric symptoms: a systematic review and meta-analysis. BMC Psychiatry. 2018;18(1):62. doi: 10.1186/s12888-018-1611-4
8. Kao HS, Lam SP, Kao TT. Chinese calligraphy handwriting (CCH): a case of rehabilitative awakening of a coma patient after stroke. Neuropsychiatr Dis Treat. 2018;14:407-417. doi: 10.2147/NDT.S147753

# Supplementary material 3

Table 1. Characteristics of 39 studies included in this study

| Author, year | Age (E/C) | Country | Male (%) | Sample size (E/C) | Diagnose | Interventions | Therapy duration | Outcome and measurement |
| --- | --- | --- | --- | --- | --- | --- | --- | --- |
| Sung et al., 2012 | 81.73±9.14/  79.50±8.76 | China | 65.8 | 27/28 | Dementia | E: Music therapy  C: Usual care | 6 weeks | Anxiety: RAID |
| Sung et al., 2010 | 78.10±7.15/  82.65±7.41 | China | 55.8 | 29/23 | Dementia | E: Music therapy  C: Usual care | 6 weeks | Anxiety: RAID |
| Pedrinolla et al., 2019 | 76.40±4.30/  78.60±4.70 | Italy | 25.8 | 82/81 | AD | E: Horticultural therapy  C: Usual care | 6 months | Cognitive function: MMSE  Activity of daily living: BI  Agitation behavior: NPI |
| Pongan et al., 2017 | 78.80±7.43/  80.20±5.71 | France | 33.9 | 31/28 | AD | E: Music therapy  C: Painting therapy | 12 weeks | Depression: GDS |
| Giovagnoli et al., 2018 | 74.30±5.70/ 72.00±7.30 | Italy | 31.1 | 23/22 | AD | E: Music therapy  C: Usual care | 24 weeks | Cognitive function: MMSE  Agitation behavior: NPI |
| Gómez-Gallego et al., 2021 | 78.67±5.73/ 80.02±5.78 | Spain | 41.9 | 21/41 | AD | E: Music therapy  C: Usual care | 12 weeks | Cognitive function: MMSE  Activity of daily living: BI  Agitation behavior: NPI |
| Hattori et al., 2011 | 75.30±5.30/ 73.30±6.30 | Japan | 46.2 | 20/19 | AD | E: Painting therapy  C: Usual care | 12 weeks | Depression: GDS |
| Raglio et al., 2015 | 81.00±7.60/ 82.40±6.80 | Italy | 22.5 | 31/35 | Dementia | E: Music therapy  C: Usual care | 10 weeks | Agitation behavior: NPI |
| Yang et al., 2021 | 84.50 (6.00)/ 85.00 (11.00) | China | 6.3 | 16/16 | AD | E: Horticultural therapy  C: Usual care | 10 weeks | Cognitive function: MMSE  Activity of daily living: BI  Quality of life: QoL-AD |
| Guétin et al., 2009 | 85.20±6.00/ 86.90±5.20 | France | 26.7 | 15/15 | AD | E: Music therapy  C: Reading therapy | 16 weeks | Depression: GDS |
| Nakamae et al., 2014 | 84.76±6.89/ 87.16±4.57 | Japan | NR | 15/15 | Dementia | E: Reminiscence therapy  C: Usual care | 6 weeks | Cognitive function: MMSE |
| Tadaka et al., 2007 | 82.50±6.60/ 81.20±6.20 (AD)  85.30±6.30/ 83.20±6.40 (VD) | Japan | 25.0 (AD)/ 38.9 (VD) | 12 (AD)/12 (VD)/18 | AD/VD | E: Reminiscence therapy  C: Usual care | 8 weeks | Cognitive function: MMSE |
| Van Bogaert et al., 2013 | 83 (65-98)/ 85 (65-101) | Belgium | 17.1 | 41/41 | AD | E: Reminiscence therapy  C: Usual care | 4 weeks | Cognitive function: MMSE  Agitation behavior: NPI |
| Goyal et al., 2021 | 84.90±7.20/ 83.30±7.10 | Norway | 26.4 | 32/40 | Dementia | E: Reading therapy  C: Usual care | 24 weeks | Anxiety: RAID |
| Li et al., 2020 | 83.21±6.73/ 83.50±5.49 | China | 55.3 | 45/45 | AD | E: Reminiscence therapy  C: Usual care | 12 weeks | Activity of daily living: BI  Agitation behavior: NPI |
| Amieva et al., 2016 | 78.80±6.90/ 78.70±6.50 | France | 38.0 | 172/154 | AD | E: Reminiscence therapy  C: Usual care | 24 months | Agitation behavior: NPI |
| Wang et al., 2007 | 79.76±6.29/ 78.92±7.64 | China | 49.0 | 51/51 | Dementia | E: Reminiscence therapy  C: Usual care | 8 weeks | Cognitive function: MMSE |
| Pérez-Sáez et al., 2021 | 82.39±7.58/ 82.68±7.32 | Spain | 29.7 | 74/74 | AD/VD | E: Reminiscence therapy  C: Usual care | 13 weeks | Cognitive function: MMSE  Quality of life: QoL-AD |
| Wu et al., 2016 | 73.50±7.30/ 73.60±7.60 | China | 31.1 | 53/50 | Dementia | E: Reminiscence therapy  C: Usual care | 6 weeks | Cognitive function: MMSE |
| Duru Aşiret et al., 2016 | 81.83±4.87/ 82.26±5.07 | Turkey | 32.3 | 31/31 | AD | E: Reminiscence therapy  C: Usual care | 12 weeks | Cognitive function: MMSE  Depression: GDS |
| Lök et al., 2019 | NR | Turkey | 43.3 | 30/30 | AD | E: Reminiscence therapy  C: Usual care | 8 weeks | Cognitive function: MMSE  Quality of life: QoL-AD |
| İnel Manav et al., 2019 | 74.06±4.52/ 74.81±4.54 | Turkey | 56.2 | 16/16 | AD | E: Reminiscence therapy  C: Usual care | 3 months | Cognitive function: MMSE |
| Lyu et al., 2018 | 68.90±7.10/ 69.90±7.90 | China | 41.1 | 100/99 | AD | E: Music therapy  C: Usual care | 3 months | Cognitive function: MMSE  Activity of daily living: BI  Agitation behavior: NPI |
| Chu et al., 2014 | 82.00±6.80 | China | 47.0 | 49/51 | Dementia | E: Music therapy  C: Usual care | 6 weeks | Cognitive function: MMSE |
| Wang et al., 2013 | 83.00±6.91/ 82.40±6.84 | China | NR | 29/29 | Dementia | E: Reminiscence therapy  C: Usual care | 8 weeks | Cognitive function: MMSE |
| Ge et al., 2013 | 69.35±12.17 | China | 57.6 | 59/59 | AD/VD | E: Reminiscence therapy  C: Usual care | 3 months | Cognitive function: MMSE  Activity of daily living: BI |
| Yuan, 2018 | 78.56±7.73/ 80.20±6.83 | China | 38.7 | 15/15 | AD/VD | E: Calligraphy therapy  C: Usual care | 12 weeks | Cognitive function: MMSE  Quality of life: QoL-AD |
| Liu et al., 2017 | NR | China | 52.0 | 12/13 | Dementia | E: Music therapy  C: Usual care | 10 weeks | Cognitive function: MMSE |
| Ito et al., 2007 | 82.90±6.40/ 82.10±5.20 | Japan | 35.3 | 17/17 | VD | E: Reminiscence therapy  C: Usual care | 3 months | Cognitive function: MMSE |
| Giovagnoli et al., 2017 | 73.92±7.74/ 75.31±5.56 | Italy | 46.2 | 13/13 | AD | E: Music therapy  C: Usual care | 12 weeks | Cognitive function: MMSE |
| Ceccato et al., 2012 | 85.50±5.90/ 87.20±7.10 | Italy | 20.0 | 27/23 | Dementia | E: Music therapy  C: Usual care | 12 weeks | Cognitive function: MMSE  Depression: GDS |
| Elfrink et al., 2021 | 79.50±8.10/ 81.20±11.20 | Netherlands | 44.2 | 19/17 | Dementia | E: Reminiscence therapy  C: Usual care | 3 months | Agitation behavior: NPI |
| Hatakeyama et al., 2010 | 82.00±10.00/ 81.00±11.00 | Japan | 39.3 | 13/15 | Dementia | E: Reminiscence therapy  C: Usual care | 4 weeks | Agitation behavior: NPI |
| Li et al., 2016 | 78.12±7.31/ 77.31±6.49 | China | 39.2 | 26/25 | Dementia | E: Reminiscence therapy  C: Usual care | 8 weeks | Agitation behavior: NPI |
| Hong et al., 2011 | 78.30±6.30 | Korea | 6.7 | 15/15 | Dementia | E: Music therapy  C: Usual care | 16 weeks | Cognitive function: MMSE |
| Janata, 2012 | 80.90±9.60/ 81.70±7.50 | USA | NR | 19/19 | Dementia | E: Music therapy  C: Usual care | 12 weeks | Cognitive function: MMSE |
| Du et al., 2018 | 78.39 ±5.72/ 79.05±5. 84 | China | 63.9 | 31/30 | Dementia | E: Music therapy  C: Usual care | 12 weeks | Cognitive function: MMSE |
| Tang et al., 2018 | 76.39±4.86/ 75.38±4.94 | China | 50.6 | 39/38 | Dementia | E: Music therapy  C: Usual care | 12 weeks | Cognitive function: MMSE |
| Lv et al., 2014 | 68.79±7.03/ 70.42±8.41/ 69.91±7.84 | China | 31.2 | 31/31/30 | AD | E: Music/ Reading therapy  C: Usual care | 3 months | Cognitive function: MMSE  Agitation behavior: NPI |

Note: NR: No report; E: Experimental group; C: Control group; AD: Alzheimer's Disease; VD: Vascular dementia; MMSE: Mini-Mental State Examination scale; BI: Barthel index; GDS: Geriatric Depression Scale; RAID: Rating of Anxiety in Dementia scale; NPI: Neuropsychiatric Inventory scale; QoL-AD: Quality of Life in Alzheimer’s disease scale.

# Supplementary material 4

Table 2. Risk of bias for included studies

| **Author, year** | **Domain 1** | **Domain 2** | **Domain 3** | **Domain 4** | **Domain 5** | **Overall risk of bias** |
| --- | --- | --- | --- | --- | --- | --- |
| Sung et al., 2012 | SC | SC | L | SC | L | SC |
| Sung et al., 2010 | SC | SC | L | SC | L | SC |
| Pedrinolla et al., 2019 | L | L | L | L | L | L |
| Pongan et al., 2017 | SC | L | L | L | L | SC |
| Giovagnoli et al., 2018 | SC | L | L | L | L | SC |
| Gómez-Gallego et al., 2021 | SC | L | L | L | L | SC |
| Hattori et al., 2011 | SC | L | L | L | L | SC |
| Raglio et al., 2015 | SC | L | L | L | L | SC |
| Yang et al., 2021 | L | L | L | L | L | L |
| Guétin et al., 2009 | SC | L | L | L | L | SC |
| Nakamae et al., 2014 | SC | L | L | L | L | SC |
| Tadaka et al., 2007 | SC | L | L | L | L | SC |
| Van Bogaert et al., 2013 | H | L | L | L | L | H |
| Goyal et al., 2021 | SC | L | L | L | L | SC |
| Li et al., 2020 | SC | L | L | L | L | SC |
| Amieva et al., 2016 | L | L | L | L | L | L |
| Wang et al., 2007 | SC | L | L | L | L | SC |
| Pérez-Sáez et al., 2021 | L | L | L | L | L | L |
| Wu et al., 2016 | SC | SC | L | SC | L | SC |
| Duru Aşiret et al., 2016 | H | L | L | L | L | H |
| Lök et al., 2019 | L | L | L | L | L | L |
| İnel Manav et al., 2019 | SC | L | L | L | L | SC |
| Lyu et al., 2018 | L | L | L | L | L | L |
| Chu et al., 2014 | L | L | L | L | L | L |
| Wang et al., 2013 | SC | SC | L | SC | L | SC |
| Ge et al., 2013 | SC | SC | L | SC | L | SC |
| Yuan, 2018 | SC | L | L | L | L | SC |
| Liu et al., 2017 | SC | SC | L | SC | L | SC |
| Ito et al., 2007 | SC | L | L | L | L | SC |
| Giovagnoli et al., 2017 | SC | L | L | L | L | SC |
| Ceccato et al., 2012 | SC | L | L | L | L | SC |
| Elfrink et al., 2021 | SC | L | L | L | L | SC |
| Hatakeyama et al., 2010 | SC | L | L | L | L | SC |
| Li et al., 2016 | L | L | L | L | L | L |
| Hong et al., 2011 | L | L | L | L | L | L |
| Janata, 2012 | SC | L | L | L | L | SC |
| Du et al., 2018 | SC | SC | L | SC | L | SC |
| Tang et al., 2018 | SC | L | L | L | L | SC |
| Lv et al., 2014 | SC | L | L | L | L | SC |

Note: Domain 1: bias arising from the randomization process, Domain 2: bias due to deviations from intended interventions, Domain 3: bias due to missing outcome data, Domain 4: bias in measurement of the outcome, Domain 5: bias in selection of the reported result, L: low risk of bias, H: high risk of bias, SC: some concern.

# Supplementary material 5


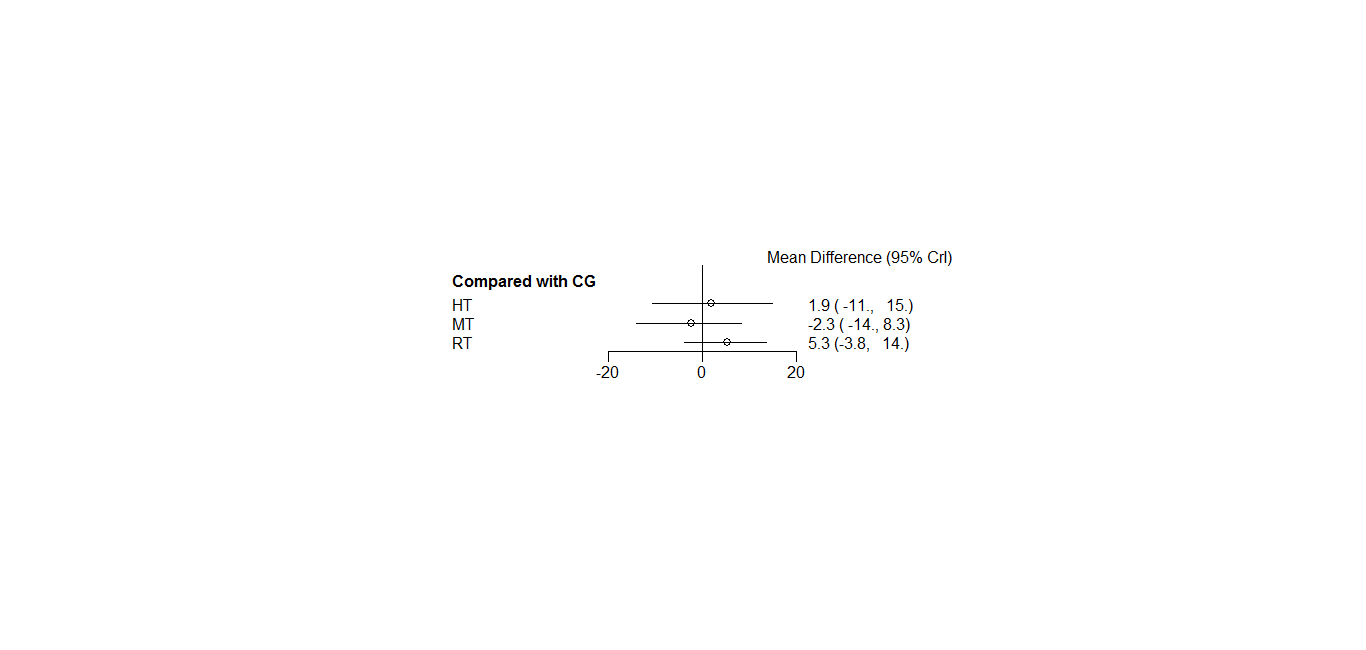


(a) Cognitive function

(b) Activity of daily living

(c) Depression


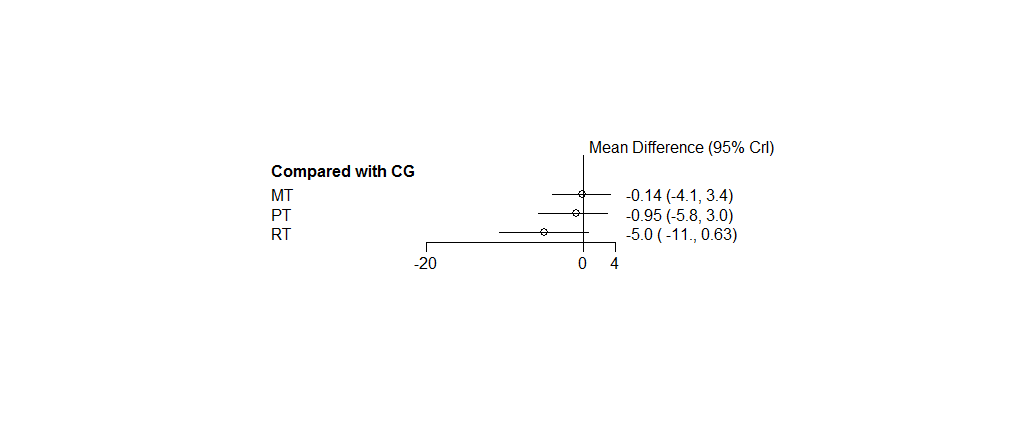


(d) Anxiety


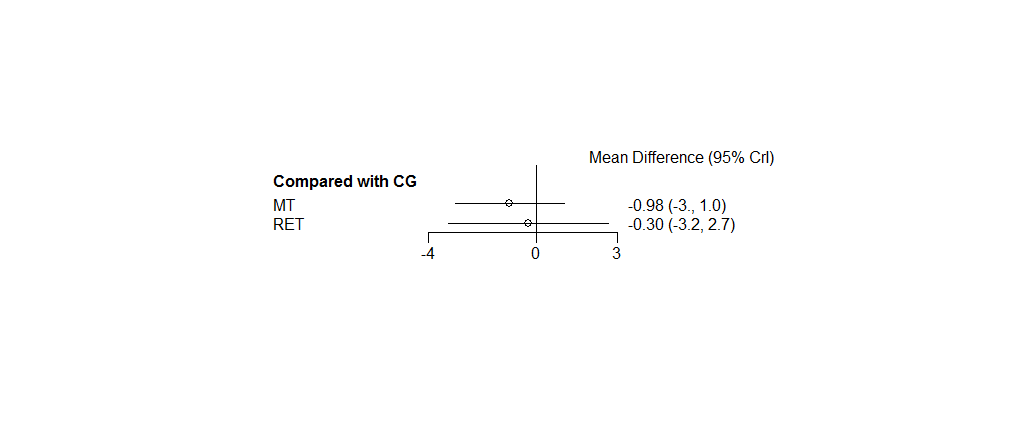


(e) Agitation behavior

(f) Quality of life


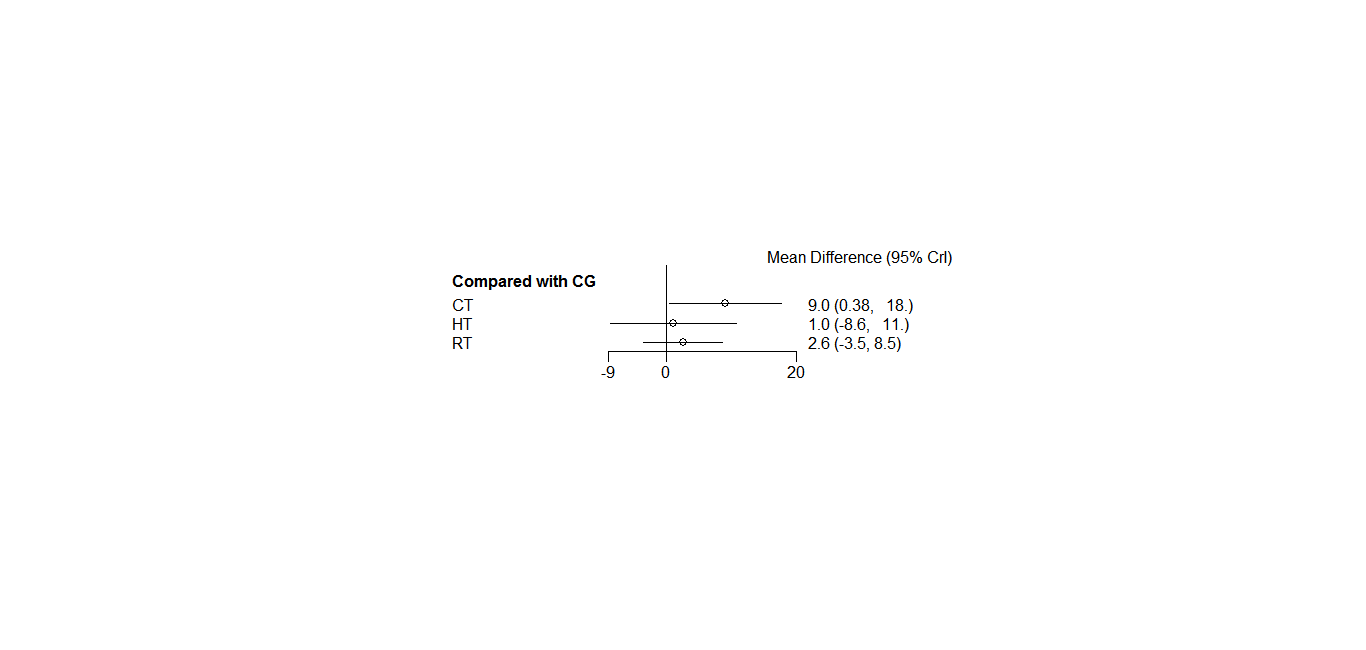

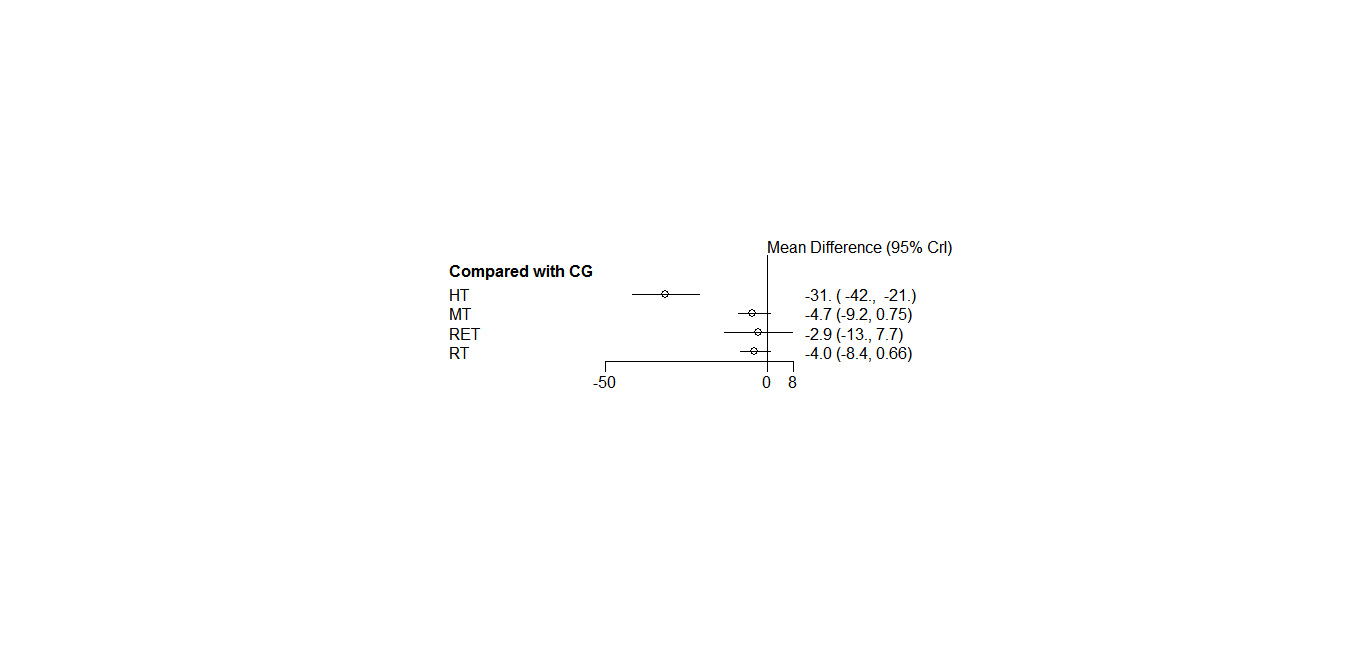

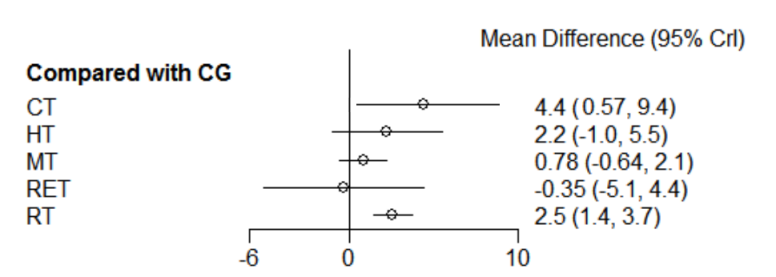


Figure 1. Forest plots of network meta-analysis results using Random Effects Model for outcomes. (a) cognitive function, (b) activity of daily living, (c) depression, (d) anxiety, (e) agitation behavior and (f) quality of life. Note: MT: music therapy; RT: reminiscence therapy; HT: horticultural therapy; CT: calligraphy therapy; RET: reading therapy; PT: painting therapy; CG: control group with usual care; CrI: credible interval.

# Supplementary material 6

Table 3. The SUCRA values of different interventions

| Outcomes | CG | CT | HT | MT | RET | RT |
| --- | --- | --- | --- | --- | --- | --- |
| Cognitive function | 0.16 | 0.87 | 0.62 | 0.37 | 0.23 | 0.73 |
| Agitation behavior | 0.08 | - | 0.99 | 0.55 | 0.38 | 0.49 |
| Quality of life | 0.18 | 0.95 | 0.35 | - | - | 0.53 |

Note: MT: music therapy; RT: reminiscence therapy; DT: dancing therapy; HT: Horticultural therapy; CT: Calligraphy therapy; RET: Reading therapy; PT: Painting therapy; CG: Control group with usual care.

# Supplementary Material 7. Lists of included studies for sensitivity analysis

**Cognitive function**

1. Pedrinolla A, Tamburin S, Brasioli A, Sollima A, Fonte C, Muti E, et al. An Indoor Therapeutic Garden for Behavioral Symptoms in Alzheimer's Disease: A Randomized Controlled Trial. J Alzheimers Dis. 2019;71(3):813-823. doi: 10.3233/JAD-190394
2. Giovagnoli AR, Manfredi V, Schifano L, Paterlini C, Parente A, Tagliavini F. Combining drug and music therapy in patients with moderate Alzheimer's disease: a randomized study. Neurol Sci. 2018;39(6):1021-1028. doi: 10.1007/s10072-018-33 16-3
3. Gómez-Gallego M, Gómez-Gallego JC, Gallego-Mellado M, García-García J. Comparative Efficacy of Active Group Music Intervention versus Group Music Listening in Alzheimer's Disease. Int J Environ Res Public Health. 2021;18(15):8067. doi: 10.3390/ijerph18158067
4. Yang Y, Kwan RYC, Zhai HM, Xiong Y, Zhao T, Fang KL, Zhang HQ. Effect of horticultural therapy on apathy in nursing home residents with dementia: a pilot randomized controlled trial. Aging Ment Health. 2022;26(4):745-753. doi: 10.1080/13607863.2021.1907304
5. Nakamae T, Yotsumoto K, Tatsumi E, Hashimotoa T. Effects of Productive Activities with Reminiscence in Occupational Therapy for People with Dementia: A Pilot Randomized Controlled Study. Hong Kong Journal of Occupational Therapy. 2014;24(1):13-19.doi: 10.1016/j.hkjot.2014.01.003
6. Tadaka E, Kanagawa K. Effects of reminiscence group in elderly people with Alzheimer disease and vascular dementia in a community setting. Geriatr Gerontol Int. 2007; 7(2):167-173. doi: 10.1111/j.1447-0594.2007.00381.x
7. Van Bogaert P, Van Grinsven R, Tolson D, Wouters K, Engelborghs S, Van der Mussele S. Effects of SolCos model-based individual reminiscence on older adults with mild to moderate dementia due to Alzheimer disease: a pilot study. J Am Med Dir Assoc. 2013;14(7):528.e9-13. doi: 10.1016/j.jamda.2013.01.020
8. Wang JJ. Group reminiscence therapy for cognitive and affective function of demented elderly in Taiwan. Int J Geriatr Psychiatry. 2007;22(12):1235-40. doi: 10.1002/gps.1821
9. Pérez-Sáez E, Justo-Henriques SI, Alves Apóstolo JL. Multicenter randomized controlled trial of the effects of individual reminiscence therapy on cognition, depression and quality of life: Analysis of a sample of older adults with Alzheimer's disease and vascular dementia. Clin Neuropsychol. 2021:1-22. doi: 10.1080/13854046.2021.1871962
10. Wu LF, Koo M. Randomized controlled trial of a six-week spiritual reminiscence intervention on hope, life satisfaction, and spiritual well-being in elderly with mild and moderate dementia. Int J Geriatr Psychiatry. 2016;31(2):120-7. doi: 10.1002/gps.4300
11. Duru Aşiret G, Kapucu S. The Effect of Reminiscence Therapy on Cognition, Depression, and Activities of Daily Living for Patients With Alzheimer Disease. J Geriatr Psychiatry Neurol. 2016;29(1):31-7. doi: 10.1177/0891988715598233
12. Lök N, Bademli K, Selçuk-Tosun A. The effect of reminiscence therapy on cognitive functions, depression, and quality of life in Alzheimer patients: Randomized controlled trial. Int J Geriatr Psychiatry. 2019;34(1):47-53. doi: 10.1002/gps.4980
13. İnel Manav A, Simsek N. The Effect of Reminiscence Therapy With Internet-Based Videos on Cognitive Status and Apathy of Older People With Mild Dementia. J Geriatr Psychiatry Neurol. 2019;32(2):104-113. doi: 10.1177/0891988718819864
14. Lyu J, Zhang J, Mu H, Li W, Champ M, Xiong Q, et al. The Effects of Music Therapy on Cognition, Psychiatric Symptoms, and Activities of Daily Living in Patients with Alzheimer's Disease. J Alzheimers Dis. 2018;64(4):1347-1358. doi: 10.3233/JAD-180183
15. Chu H, Yang CY, Lin Y, Ou KL, Lee TY, O'Brien AP, et al. The impact of group music therapy on depression and cognition in elderly persons with dementia: a randomized controlled study. Biol Res Nurs. 2014;16(2):209-17. doi: 10.1177/1099800413485410
16. Wang LY, Shen CZ. Intervention practice of senile dementia based on life story book.Chinese Journal of Gerontology. 2013;33(21):5239-5241. (in Chinese)
17. Ge ZX, Deng XL. Observation on rehabilitation effect of memory therapy in senile dementia patients.Journal of Nursing(China). 2013;20(03):63-65. (in Chinese) doi: 10.16460/j.issn1008-9969.2013.03.025.
18. Yuan LX. Effect of arithmetic or drawing writing on communication and cognitive function in patients with mild to moderate dementia. Guangzhou Medical University: Master Thesis, 2018. (in Chinese)
19. Ito T, Meguro K, Akanuma K, Ishii H, Mori E. A randomized controlled trial of the group reminiscence approach in patients with vascular dementia. Dement Geriatr Cogn Disord. 2007;24(1):48-54. doi: 10.1159/000103631
20. Giovagnoli AR, Manfredi V, Parente A, Schifano L, Oliveri S, Avanzini G. Cognitive training in Alzheimer's disease: a controlled randomized study. Neurol Sci. 2017;38(8):1485-1493. doi: 10.1007/s10072-017-3003-9
21. Ceccato E, Vigato G, Bonetto C, Bevilacqua A, Pizziolo P, Crociani S, Zanfretta E, Pollini L, Caneva PA, Baldin L, Frongillo C, Signorini A, Demoro S, Barchi E. STAM protocol in dementia: a multicenter, single-blind, randomized, and controlled trial. Am J Alzheimers Dis Other Demen. 2012;27(5):301-10. doi: 10.1177/1533317512452038
22. Choi H M J . Songwriting oriented activities improve the cognitive functions of the aged with dementia. The Arts in Psychotherapy.2011. doi: 10.1016/j.aip.2011.07.002
23. Janata, P. Effects of Widespread and Frequent Personalized Music Programming on Agitation and Depression in Assisted Living Facility Residents With Alzheimer-Type Dementia. Music & Medicine. 2012;4(1):8-15. doi: 10.1177/1943862111430509
24. Du YQ, Zhao X. Effect of music nursing intervention on agitation behavior and cognitive function in patients with moderate and mild senile dementia. Chinese Journal of Geriatric Care. 2018;16(04):128-130. (in Chinese)
25. Tang Q, Zhou Y, Yang S, Thomas WKS, Smith GD, Yang Z, et al. Effect of music intervention on apathy in nursing home residents with dementia. Geriatr Nurs. 2018;39(4):471-476. doi: 10.1016/j.gerinurse.2018.02.003
26. Lyv JH, Gao T, Li M, Xie LJ, Li WJ, Jin WY, et al. The effect of music therapy on memory, language and psychological symptoms of patients with mild Alzheimer’s disease .Chinese Journal of Neurology. 2014;47(12):831-835. (in Chinese)

**Activity of daily living**

1. Pedrinolla A, Tamburin S, Brasioli A, Sollima A, Fonte C, Muti E, et al. An Indoor Therapeutic Garden for Behavioral Symptoms in Alzheimer's Disease: A Randomized Controlled Trial. J Alzheimers Dis. 2019;71(3):813-823. doi: 10.3233/JAD-190394
2. Gómez-Gallego M, Gómez-Gallego JC, Gallego-Mellado M, García-García J. Comparative Efficacy of Active Group Music Intervention versus Group Music Listening in Alzheimer's Disease. Int J Environ Res Public Health. 2021;18(15):8067. doi: 10.3390/ijerph18158067
3. Li M, Lyu JH, Zhang Y, Gao ML, Li R, Mao PX, et al. Efficacy of Group Reminiscence Therapy on Cognition, Depression, Neuropsychiatric Symptoms, and Activities of Daily Living for Patients With Alzheimer Disease. J Geriatr Psychiatry Neurol. 2020;33(5):272-281. doi: 10.1177/0891988719882099
4. Lyu J, Zhang J, Mu H, Li W, Champ M, Xiong Q, et al. The Effects of Music Therapy on Cognition, Psychiatric Symptoms, and Activities of Daily Living in Patients with Alzheimer's Disease. J Alzheimers Dis. 2018;64(4):1347-1358. doi: 10.3233/JAD-180183
5. Ge ZX, Deng XL. Observation on rehabilitation effect of memory therapy in senile dementia patients.Journal of Nursing(China). 2013;20(03):63-65. (in Chinese) doi: 10.16460/j.issn1008-9969.2013.03.025.

**Depression**

1. Pongan E, Tillmann B, Leveque Y, Trombert B, Getenet JC, et al. Can Musical or Painting Interventions Improve Chronic Pain, Mood, Quality of Life, and Cognition in Patients with Mild Alzheimer's Disease? Evidence from a Randomized Controlled Trial. J Alzheimers Dis. 2017;60(2):663-677. doi: 10.3233/JAD-170410
2. Hattori H, Hattori C, Hokao C, Mizushima K, Mase T. Controlled study on the cognitive and psychological effect of coloring and drawing in mild Alzheimer's disease patients. Geriatr Gerontol Int. 2011;11(4):431-7. doi: 10.1111/j.1447-0594.2011.00698.x
3. Duru Aşiret G, Kapucu S. The Effect of Reminiscence Therapy on Cognition, Depression, and Activities of Daily Living for Patients With Alzheimer Disease. J Geriatr Psychiatry Neurol. 2016;29(1):31-7. doi: 10.1177/0891988715598233
4. Ceccato E, Vigato G, Bonetto C, Bevilacqua A, Pizziolo P, Crociani S, Zanfretta E, Pollini L, Caneva PA, Baldin L, Frongillo C, Signorini A, Demoro S, Barchi E. STAM protocol in dementia: a multicenter, single-blind, randomized, and controlled trial. Am J Alzheimers Dis Other Demen. 2012;27(5):301-10. doi: 10.1177/1533317512452038

**Anxiety**

1. Sung HC, Lee WL, Li TL, Watson R. A group music intervention using percussion instruments with familiar music to reduce anxiety and agitation of institutionalized older adults with dementia. Int J Geriatr Psychiatry. 2012;27(6):621-7. doi: 10.1002/gps.2761
2. Goyal AR, Engedal K, Benth JŠ, Strøm BS. Effects of the Sonas Program on Anxiety and Depression in Nursing Home Residents with Dementia: A 6-Month Randomized Controlled Trial. Dement Geriatr Cogn Dis Extra. 2021;11(2):151-158. doi: 10.1159/000516804

**Agitation behavior**

1. Pedrinolla A, Tamburin S, Brasioli A, Sollima A, Fonte C, Muti E, et al. An Indoor Therapeutic Garden for Behavioral Symptoms in Alzheimer's Disease: A Randomized Controlled Trial. J Alzheimers Dis. 2019;71(3):813-823. doi: 10.3233/JAD-190394
2. Giovagnoli AR, Manfredi V, Schifano L, Paterlini C, Parente A, Tagliavini F. Combining drug and music therapy in patients with moderate Alzheimer's disease: a randomized study. Neurol Sci. 2018;39(6):1021-1028. doi: 10.1007/s10072-018-33 16-3
3. Gómez-Gallego M, Gómez-Gallego JC, Gallego-Mellado M, García-García J. Comparative Efficacy of Active Group Music Intervention versus Group Music Listening in Alzheimer's Disease. Int J Environ Res Public Health. 2021;18(15):8067. doi: 10.3390/ijerph18158067
4. Raglio A, Bellandi D, Baiardi P, Gianotti M, Ubezio MC, Zanacchi E, et al. Effect of Active Music Therapy and Individualized Listening to Music on Dementia: A Multicenter Randomized Controlled Trial. J Am Geriatr Soc. 2015;63(8):1534-9. doi: 10.1111/jgs.13558
5. Van Bogaert P, Van Grinsven R, Tolson D, Wouters K, Engelborghs S, Van der Mussele S. Effects of SolCos model-based individual reminiscence on older adults with mild to moderate dementia due to Alzheimer disease: a pilot study. J Am Med Dir Assoc. 2013;14(7):528.e9-13. doi: 10.1016/j.jamda.2013.01.020
6. Li M, Lyu JH, Zhang Y, Gao ML, Li R, Mao PX, et al. Efficacy of Group Reminiscence Therapy on Cognition, Depression, Neuropsychiatric Symptoms, and Activities of Daily Living for Patients With Alzheimer Disease. J Geriatr Psychiatry Neurol. 2020;33(5):272-281. doi: 10.1177/0891988719882099
7. Amieva H, Robert PH, Grandoulier AS, Meillon C, De Rotrou J, Andrieu S, et al. Group and individual cognitive therapies in Alzheimer's disease: the ETNA3 randomized trial. Int Psychogeriatr. 2016;28(5):707-17. doi: 10.1017/S1041610215001830
8. Lyu J, Zhang J, Mu H, Li W, Champ M, Xiong Q, et al. The Effects of Music Therapy on Cognition, Psychiatric Symptoms, and Activities of Daily Living in Patients with Alzheimer's Disease. J Alzheimers Dis. 2018;64(4):1347-1358. doi: 10.3233/JAD-180183
9. Elfrink TR, Ullrich C, Kunz M, Zuidema SU, Westerhof GJ. The Online Life Story Book: A randomized controlled trial on the effects of a digital reminiscence intervention for people with (very) mild dementia and their informal caregivers. PLoS One. 2021;16(9):e0256251. doi: 10.1371/journal.pone.0256251
10. Li M, Lyu JH, Gao ML, Hao ZH, Ma L, Li WJ. The effect of reminiscence therapy on behavioral and psychological symptoms in people with mild and moderate dementia. Beijing Medical Journal. 2016;38(10):999-1002. (in Chinese) doi: 10.15932/j.0253-9713.2016.10.008
11. Lyv JH, Gao T, Li M, Xie LJ, Li WJ, Jin WY, et al. The effect of music therapy on memory, language and psychological symptoms of patients with mild Alzheimer’s disease .Chinese Journal of Neurology. 2014;47(12):831-835. (in Chinese)

**Quality of life**

1. Yang Y, Kwan RYC, Zhai HM, Xiong Y, Zhao T, Fang KL, Zhang HQ. Effect of horticultural therapy on apathy in nursing home residents with dementia: a pilot randomized controlled trial. Aging Ment Health. 2022;26(4):745-753. doi: 10.1080/13607863.2021.1907304
2. Pérez-Sáez E, Justo-Henriques SI, Alves Apóstolo JL. Multicenter randomized controlled trial of the effects of individual reminiscence therapy on cognition, depression and quality of life: Analysis of a sample of older adults with Alzheimer's disease and vascular dementia. Clin Neuropsychol. 2021:1-22. doi: 10.1080/13854046.2021.1871962
3. Yuan LX. Effect of arithmetic or drawing writing on communication and cognitive function in patients with mild to moderate dementia. Guangzhou Medical University: Master Thesis, 2018. (in Chinese)

# Supplementary material 8. Results of sensitivity analysis


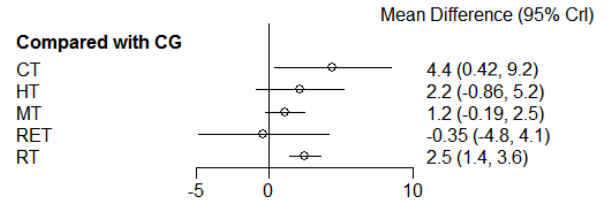


(a) Cognitive function

(b) Activity of daily living

(c) Depression

(d) Anxiety

(e) Agitation behavior

(f) Quality of life


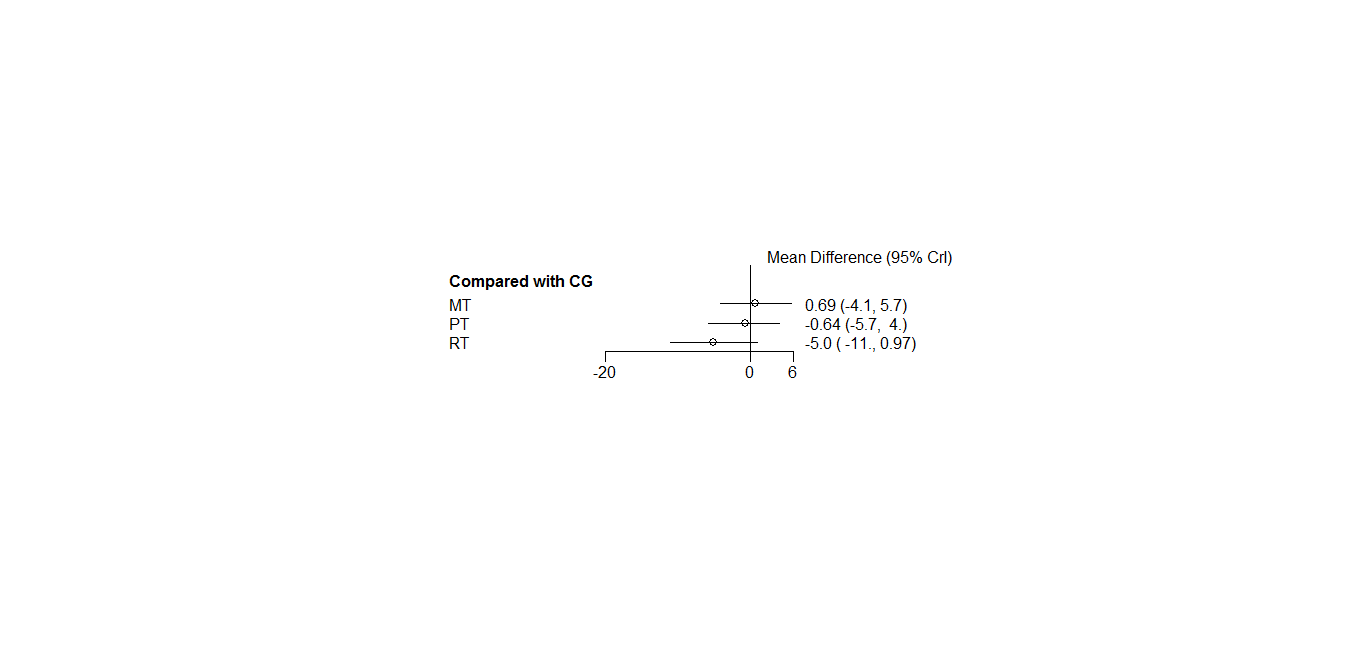

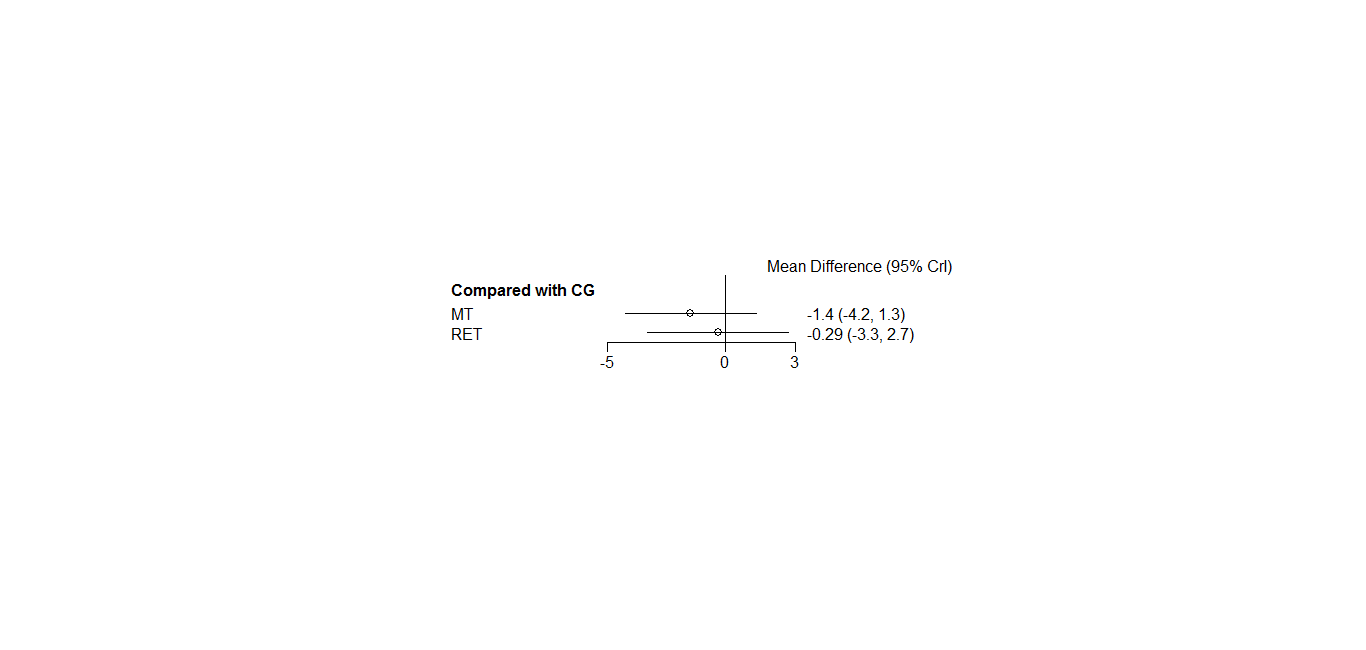

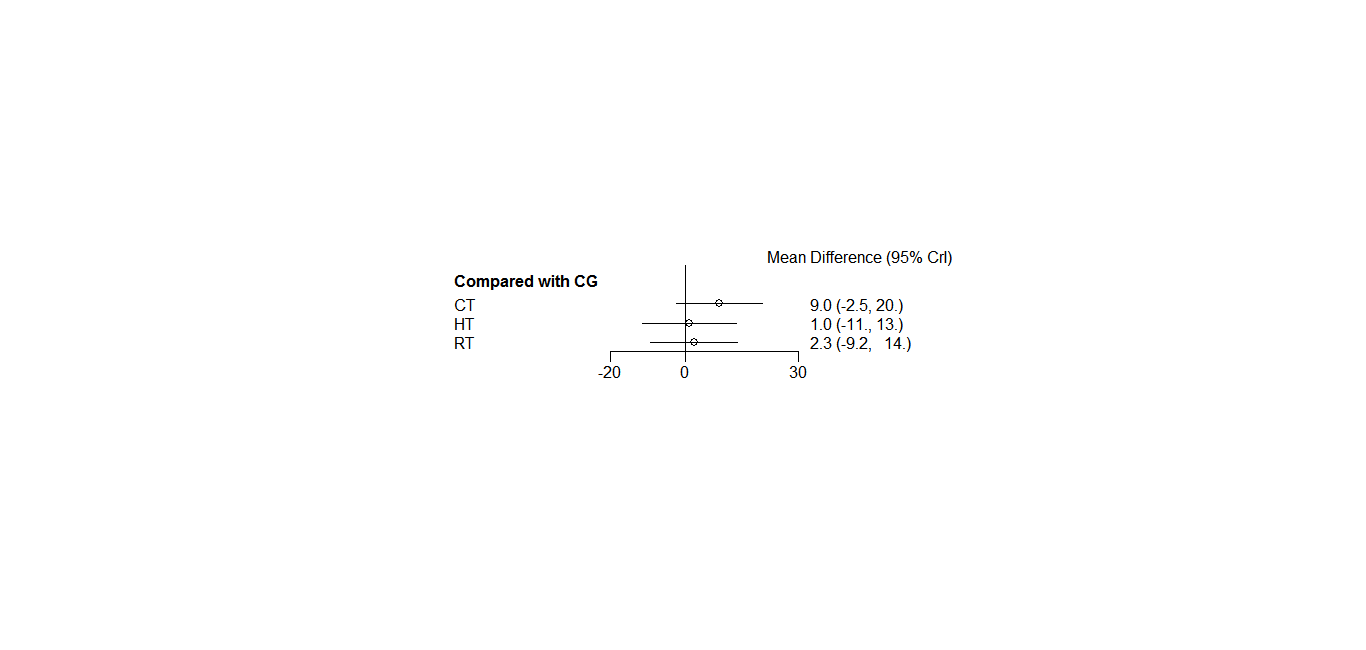

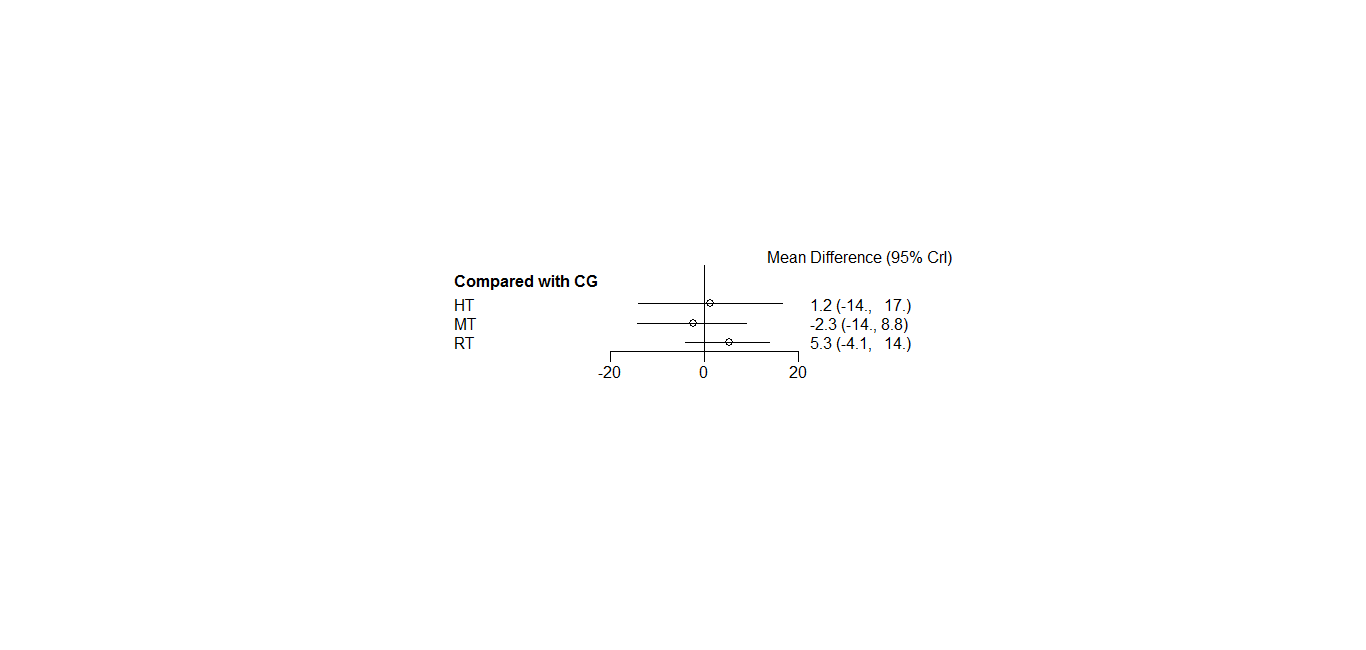

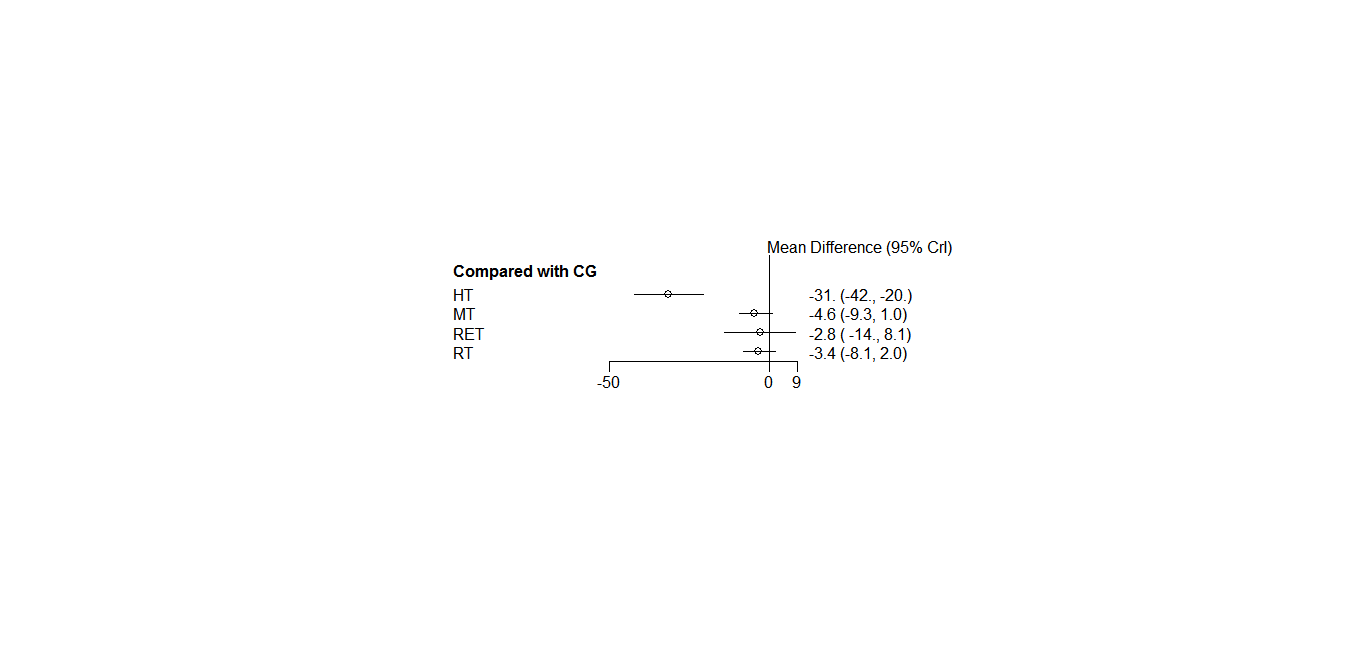


Figure 2. Forest plots for sensitivity analysis. (a) cognitive function, (b) activity of daily living, (c) depression, (d) anxiety, (e) agitation behavior and (f) quality of life. Note: MT: music therapy; RT: reminiscence therapy; HT: horticultural therapy; CT: calligraphy therapy; RET: reading therapy; PT: painting therapy; CG: control group with usual care; CrI: credible interval.

Table 4. League tables for sensitivity analysis of different interventions on each outcome

1. Cognitive function

| **CG** |  |  |  |  |  |
| --- | --- | --- | --- | --- | --- |
| **-4.40 (-9.19, -0.42)** | **CT** |  |  |  |  |
| -2.16 (-5.23, 0.86) | 2.23 (-3.46, 7.89) | **HT** |  |  |  |
| -1.17 (-2.52, 0.19) | 3.23 (-1.75, 8.23) | 0.99 (-2.32, 4.36) | **MT** |  |  |
| 0.35 (-4.15, 4.83) | 4.74 (-1.79, 11.28) | 2.51 (-2.89, 7.94) | 1.51 (-3.18, 6.17) | **RET** |  |
| **-2.54 (-3.60, -1.44)** | 1.86 (-3.07, 6.79) | -0.37 (-3.56, 2.90) | **-1.37 (-3.10, -0.36)** | -2.88 (-7.50, 1.74) | **RT** |

1. Activity of daily living

| **CG** |  |  |  |
| --- | --- | --- | --- |
| -1.22(-16.52, 14.05) | **HT** |  |  |
| 2.32 (-8.80, 14.21) | 3.53(-15.23, 22.99) | **MT** |  |
| -5.26(-13.78, 4.11) | -4.06 (-21.36, 13.96) | -7.55 (-22.12, 6.92) | **RT** |

1. Depression

| **CG** |  |  |  |
| --- | --- | --- | --- |
| -0.69 (-5.72, 4.12) | **MT** |  |  |
| 0.64 (-3.97, 5.74) | 1.35 (-3.37, 6.69) | **PT** |  |
| 5.02 (-0.97, 10.98) | 5.72 (-1.93, 13.54) | 4.38 (-3.512, 11.85) | **RT** |

1. Anxiety

| **CG** |  |  |
| --- | --- | --- |
| 1.45 (-1.32, 4.24) | **MT** |  |
| 0.29 (-2.70, 3.29) | -1.16 (-5.24, 2.92) | **RET** |

1. Agitation behavior

| **CG** |  |  |  |  |
| --- | --- | --- | --- | --- |
| **31.28 (20.46, 42.15)** | **HT** |  |  |  |
| 4.64 (-1.01, 9.31) | **-26.64 (-39.12, -15.21)** | **MT** |  |  |
| 2.80 (-8.11, 13.80) | **-28.48 (-43.81, -13.00)** | -1.84 (-13.40, 10.83) | **RET** |  |
| 3.37 (-2.01, 8.11) | **-27.93 (-40.16, -16.35)** | -1.28 (-8.26, 6.12) | 0.55 (-11.87, 12.30) | **RT** |

1. Quality of life

| **CG** |  |  |  |
| --- | --- | --- | --- |
| -9.01(-20.47, 2.50) | **CT** |  |  |
| -1.03 (-13.40, 11.41) | 7.97 (-8.95, 24.94) | **HT** |  |
| -2.33 (-13.76, 9.17) | 6.68 (-9.60, 23.04) | -1.30 (-18.21, 15.57) | **RT** |

Note: MT: music therapy; RT: reminiscence therapy; HT: horticultural therapy; CT: calligraphy therapy; RET: reading therapy; PT: painting therapy; CG: control group with usual care.

Table 5. The SUCRA values of different interventions for sensitivity analysis

| Outcomes | CG | CT | HT | MT | RET | RT |
| --- | --- | --- | --- | --- | --- | --- |
| Cognitive function | 0.14 | 0.87 | 0.62 | 0.42 | 0.21 | 0.73 |
| Agitation behavior | 0.10 | - | 0.99 | 0.57 | 0.39 | 0.45 |
| Quality of life | 0.18 | 0.89 | 0.38 | - | - | 0.49 |

Note: MT: music therapy; RT: reminiscence therapy; HT: Horticultural therapy; CT: Calligraphy therapy; RET: Reading therapy; PT: Painting therapy; CG: Control group with usual care.
